# Markers of epidemiological success of methicillin-resistant *Staphylococcus aureus* isolates in European populations

**DOI:** 10.1016/j.cmi.2023.05.015

**Published:** 2023-09

**Authors:** Valérie O. Baede, Arya Gupta, Gwenan M. Knight, Leo M. Schouls, Ken Laing, Mehri Tavakol, Anaïs Barray, Sake J. de Vlas, Anneke S. de Vos, Antoni P.A. Hendrickx, Madeeha Khan, Mirjam E. Kretzschmar, Willem J.B. van Wamel, Gérard Lina, Francois Vandenesch, Margreet C. Vos, Adam A. Witney, Jean-Philippe Rasigade, Jodi A. Lindsay

**Affiliations:** 1) Department of Medical Microbiology and Infectious Diseases, Erasmus MC University Medical Center Rotterdam, Rotterdam, the Netherlands; 2) Institute for Infection and Immunity, St George's, University of London, London, United Kingdom; 3) AMR Centre, Centre for Mathematical Modelling of Infectious Diseases, Infectious Disease Epidemiology, London School of Hygiene and Tropical Medicine, London, United Kingdom; 4) Centre for Infectious Disease Control, National Institute for Public Health and the Environment (RIVM), Bilthoven, the Netherlands; 5) CIRI, Centre International de Recherche en Infectiologie, Inserm U1111, Université Lyon 1, Ecole Normale Supérieure de Lyon, Lyon, France; 6) Centre National de Référence des Staphylocoques, Institut des Agent Infectieux, Hôpital de La Croix Rousse, Hospices Civils de Lyon, Lyon, France; 7) Department of Public Health, Erasmus MC, University Medical Center Rotterdam, Rotterdam, the Netherlands; 8) Julius Center for Health Sciences and Primary Care, University Medical Center Utrecht, Utrecht University, Utrecht, the Netherlands

**Keywords:** Antimicrobial resistance, Antimicrobial usage, Epidemiology, MRSA, Success, Whole-genome sequencing

## Abstract

**Objectives:**

Methicillin-resistant *Staphylococcus aureus* (MRSA) infections impose a considerable burden on health systems, yet there is remarkable variation in the global incidence and epidemiology of MRSA. The MACOTRA consortium aimed to identify bacterial markers of epidemic success of MRSA isolates in Europe using a representative MRSA collection originating from France, the Netherlands and the United Kingdom.

**Methods:**

Operational definitions of success were defined in consortium meetings to compose a balanced strain collection of successful and sporadic MRSA isolates. Isolates were subjected to antimicrobial susceptibility testing and whole-genome sequencing; genes were identified and phylogenetic trees constructed. Markers of epidemiological success were identified using genome-based time-scaled haplotypic density analysis and linear regression. Antimicrobial usage data from ESAC-Net was compared with national MRSA incidence data.

**Results:**

Heterogeneity of MRSA isolate collections across countries hampered the use of a unified operational definition of success; therefore, country-specific approaches were used to establish the MACOTRA strain collection. Phenotypic antimicrobial resistance varied within related MRSA populations and across countries. In time-scaled haplotypic density analysis, fluoroquinolone, macrolide and mupirocin resistance were associated with MRSA success, whereas gentamicin, rifampicin and trimethoprim resistance were associated with sporadicity. Usage of antimicrobials across 29 European countries varied substantially, and β-lactam, fluoroquinolone, macrolide and aminoglycoside use correlated with MRSA incidence.

**Discussion:**

Our results are the strongest yet to associate MRSA antibiotic resistance profiles and antibiotic usage with the incidence of infection and successful clonal spread, which varied by country. Harmonized isolate collection, typing, resistance profiling and alignment with antimicrobial usage over time will aid comparisons and further support country-specific interventions to reduce MRSA burden.

## Introduction

Antimicrobial resistance (AMR) is considered to be the greatest threat to the future of modern medicine, and methicillin-resistant *Staphylococcus aureus* (MRSA) is estimated to be the most common cause of AMR-associated deaths globally and second in Europe [1,2]. MRSA carriage is a risk factor for subsequent infection [[3], [4], [5]]. MRSA are resistant to virtually the full spectrum of β-lactam antimicrobials because of the *mecA*-carrying SCC*mec* mobile element acquired on multiple occasions into different *S. aureus* genetic lineages, resulting in a range of different MRSA clones. Approximately a dozen clones, often discriminated by their different lineage (also called clonal complex [CC]) dominate the MRSA population globally. The dominant clone may differ in different geographic settings, and may even change over time [4,6,7]. Resistance to almost all classes of antimicrobials can be found in MRSA, although fully drug-resistant isolates are rare. Furthermore, the incidence of MRSA infection can vary widely between countries, but the reasons for these geographical differences are poorly understood.

Each country has developed distinct strategies for collecting, testing, typing and reporting of MRSA isolates [8]. This variation in approaches obstructs comparisons and proper aggregation of data and therefore thorough epidemiological analysis on an international level and could potentially hide or over-estimate common markers of successful clones across geographic settings. The implementation of whole-genome sequencing analysis can aid international data comparison and can be used to search for genotypic markers of successful isolates.

France, the UK and the Netherlands represent countries with very differing incidences of MRSA infection, recently estimated at 48.13, 10.65 and 1.47 per 100 000 population, respectively [2]. Each country is dominated by different clones [[9], [10], [11]], with community isolates rare in the UK, and livestock isolates more prevalent in the Netherlands. Here, we used two approaches to identify epidemiologically successful isolates in each country and searched for genetic and AMR biomarkers of success. Further analysis of resistance markers revealed associations between the usage of specific antimicrobial classes and MRSA incidence across 29 European countries, identifying potential targets for stewardship interventions.

## Methods

Full details are in the Supplementary Methods section.

### Operational definition and collection of successful and sporadic (unsuccessful) isolates

Collaborators from each country, France, the Netherlands and the UK in the MACOTRA consortium (Combating MRSA; increasing our understanding of transmission success will lead to better control of MRSA), identified their country-specific epidemiological characteristics of MRSA success over time, which included incidence of infection and local typing methods. Where common criteria across countries could be identified this was used. The success selection criteria were designed to not be biased by lineage, AMR profile or any other biomarker.

### Whole-genome sequencing and epidemiological clustering

Whole-genome sequencing used the Illumina MiSeq platform. Sequence reads were aligned to reference genomes (RefSeq NC_002952 [CC30], NC_017763 [CC22], NC_002745 [CC5]). Phylogenetic reconstruction was performed using IQ-Tree version 2.0.3. Genomes were also assembled using Shovill v1.0.9 and resistance genes were identified using Abricate and the Comprehensive Antibiotic Resistance Database, and virulence genes were identified using the Virulence Factor Database [12,13]. Genes associated with operational success were compared using χ2 tests at a 5% significance threshold.

### Antimicrobial susceptibility testing

EUCAST disk diffusion methodology [14] was used to test for sensitivity to 14 antibiotics using Oxoid disks (Basingstoke, UK). The distribution of phenotypic resistance and successful isolates in each country was compared using χ2 tests at a 5% significance threshold.

### Time-scaled haplotypic density analysis

We used the time-scaled haplotypic density (THD) method to examine the factors predicting the epidemic success of MRSA based on genome sequences [[15], [16], [17]]. THD assigned relative indices of epidemic success, over a defined time period of 5 years, to each isolate in the dataset, on the basis of the branching density and distribution of genetic distances separating it from other isolates. Potential predictors of success, such as antimicrobial drug resistance patterns, were identified using linear regression models with THD indices as the response variable.

Genome-wide association studies (GWAS) were performed using pyseer [18], using kmer and unitig approaches utilizing the Shovill-derived denovo-assembled genome sequences. Genes associated with THD success and resistance phenotypes were also determined using pyseer following kmer- and uniting-based approaches [18].

### AMR and antimicrobial usage across Europe

The sum of estimated incidence of all infection types caused by MRSA was used to give an overall MRSA annual incidence per 100 000 population data across 29 European countries [2]. This was based on EARS-Net data adjusted for coverage and usage of diagnostics for the year 2015 [2]. Antimicrobial consumption data for 2015 were from ESAC-Net (ecdc.europa.eu) and expressed as a defined daily dose per 1000 inhabitants per day. The data for some countries were split into community and healthcare usage and sourced from national sales and reimbursement data. We tested both linear (shown here) and exponential trend associations (in Supplementary Methods), with an F test for significance: all data management and analysis is provided in Supplementary 2 and as a code in a GitHub repository: https://github.com/gwenknight/mrsa_inf_abx.

## Results

### Operational definition of epidemiologically successful and sporadic isolates

National strategies for collecting, typing and reporting MRSA isolates in each country were found to be markedly different [8], necessitating some variation in inclusion criteria between countries (Supplementary 1). AMR-resistance profiles were not used as selection criteria.

Representative isolates from France were selected from the collection of the French National Reference Centre for Staphylococcus, Lyon, France, and previously typed using DNA array hybridization [19,20]. Eight representative CC were chosen and CC subtype clusters totalling >25% of the CC size were labelled ‘successful’, whereas examples totalling <25% were labelled ‘sporadic’.

In the Netherlands, mandatory surveillance of all clinical and colonization MRSA isolates is combined with epidemiological data and typing by multiple-locus variable number-tandem repeat analysis at the Dutch National Institute for Public Health and the Environment. Seven representative clonal types aligned with CCs were chosen, and random isolates from the most-prevalent and least-prevalent multiple-locus variable number-tandem repeat analysis types with each clone were chosen as successful and sporadic isolates, respectively. In addition, some isolates from populations involved in known outbreaks but from a different type as causing the outbreak were included as sporadic isolates.

In the UK, an isolate collection from St George's University Hospital Trust, London, that documented a shift in the dominant clones over time was utilized [9]. CC30 and CC22 account for most UK isolates and whole-genome multi-locus sequence typing phylogenetic trees were constructed and clusters with <15 whole-genome multi-locus sequence typing allelic differences were defined as successful. Examples of those that did not cluster were defined as sporadic, along with examples from another six CCs.

### Phylogenetically related isolates were identified across the three countries but differed in dominance and success

**Fig. 1:**
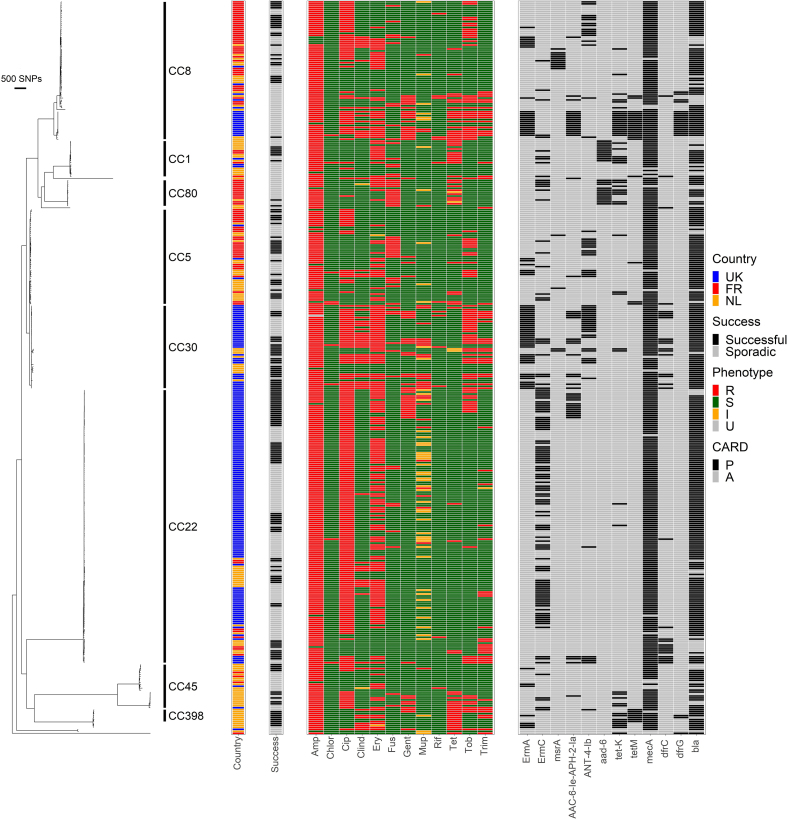
Phylogenetic tree of the collection showing CCs, country and operational success. Two panels aligned to the tree show the Resistant (R), Susceptible (S), Intermediate (I) and Unknown (U) status of each drug for each isolate, and the Presence (P) and Absence (A) of known resistance genes from the Comprehensive Antibiotic Resistance Database. Further details of the isolates are in Table S1. CC, clonal complex.

Sequence data were submitted to the European Nucleotide Archive database with accession number PRJEB47238. Phylogenetic trees of 157 successful and 221 sporadic MRSA clearly identified the different CCs (Fig. 1). Each of the three countries was dominated by isolates from different CCs. Clusters within sub-branches of each lineage were isolated suggesting dissemination into other countries (Fig. 1 and Fig. S3). Further analysis of CC22 and CC30, the most-prevalent CCs, demonstrated that transmission of an isolate to another country did not generally lead to successful localized clusters (Figs. S1 and S2).

### AMR profiles were highly variable and associated with country

**Fig. 2:**
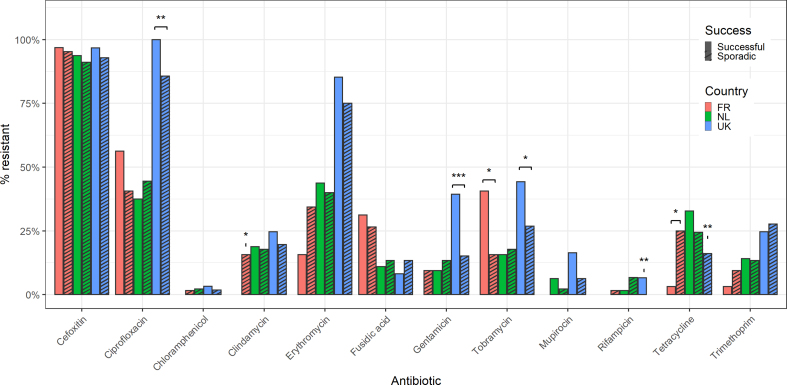
AMR resistance varied between successful and sporadic isolates within countries. AMR association is marked as∗p ≤ 0.05; ∗∗p ≤ 0.01; ∗∗∗p ≤ 0.001 by χ^2^ test. AMR, antimicrobial resistance.

AMR phenotypes and genotypes varied substantially within CCs and across CCs (Fig. 1), as well as between countries and operational definitions of success (Fig. 2). France had a higher proportion of fusidic acid resistance and a lower proportion of gentamicin, mupirocin and trimethoprim resistance. The Netherlands had a higher proportion of tetracycline resistance and a lower proportion of tobramycin resistance. The UK had a higher proportion of fluoroquinolone, erythromycin, gentamicin, mupirocin, tobramycin and trimethoprim resistance and a lower proportion of tetracycline resistance (Fig. S4).

Within countries, success in France was associated with tobramycin resistance and sporadic isolates with clindamycin and tetracycline resistance. Success in the UK was associated with ciprofloxacin, gentamicin, rifampicin and tobramycin resistance and sporadic isolates with tetracycline resistance (Fig. 2). Across the collection, successful isolates were associated with tobramycin resistance (Table S3).

### THD analysis

**Fig. 3:**
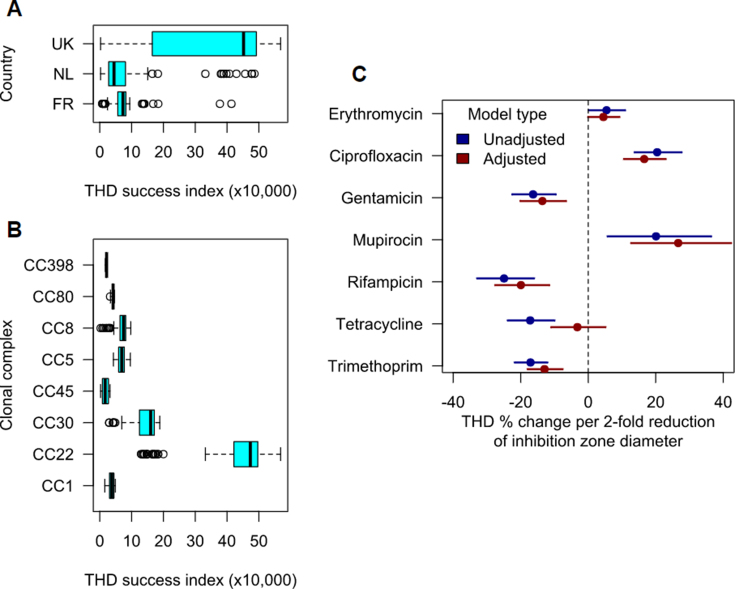
Genome-based analysis of markers of epidemiological success. Shown are the distributions of THD success indices across countries (A) and clonal complexes (B). Panel C shows pointwise estimates (dots) and 95% CI (bars) of the coefficients of regression models predicting THD indices with antimicrobial resistance, expressed as units of a two-fold reduction of inhibition zone diameters. Models were either unadjusted (blue bars) or adjusted (multivariable, red bars). In multivariable regression, THD success indices were predicted by higher ciprofloxacin, erythromycin and mupirocin resistance, and by susceptibility to gentamicin, rifampicin and trimethoprim. THD, time-scaled haplotypic density.

THD indices were assigned by genetic distance to other isolates in the collection (Table S1) reflecting the rate of transmission and selection over time [[15], [16], [17]]. THD indices were higher in the UK, suggesting that successful isolates were closely related in this country (Fig. 1, Table S1). CC22 was the most successful clone overall (Fig. 3(b)) and the dominant clone in the UK (Fig. 1, Fig. 3).

The THD and operational definitions of success did not differ significantly between successful and sporadic isolates (two-sided Mann-Whitney test, p 0.42). However, after taking into account the country and CC of each strain as random effects in a mixed-effect linear regression, operational success predicted slightly higher THD values (17.5% increase, 95% CI, 6.7–29.4%, p 0.001). These findings indicate that the operational definitions of success failed to capture the epidemic success of a given isolate among the global MRSA population, most likely because of the country-specific definitions; however, operational definitions correctly predicted epidemic success within the same lineage and country.

Then, we leveraged THD indices to examine AMR with epidemic success. We constructed univariate and multivariable models of the THD success index as a function of the inhibition zone diameters for seven antimicrobial drugs. All models were adjusted for variations across countries and CCs using random intercepts. In multivariable analysis, the THD success index correlated positively with ciprofloxacin, erythromycin and mupirocin, and negatively with gentamicin, rifampicin and trimethoprim (Fig. 3(c)).

GWAS using pyseer to identify mutations and account for lineage variation did not reveal any markers of success. Pyseer correctly identified the most common mutations associated with phenotypic resistance (Fig. S5). A search for known virulence genes using Virulence Factor Database identified *tst* (toxic shock syndrome toxin) although the prevalence was low (Table S1 and Supplementary Data).

### AMR incidence and antimicrobial usage across Europe

**Fig. 4:**
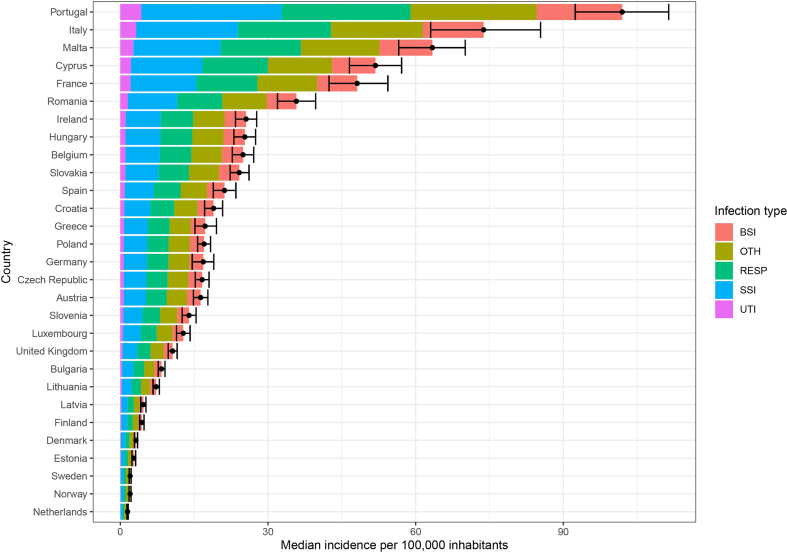
Median infection incidence in 29 European countries because of MRSA in 2015 as estimated by Cassini et al. [25] for five infection types (colours) and total (point) with 97.5% to 102.5% confidence ranges. The five infection types are bloodstream infections (BSI), urinary tract infections (UTI), respiratory tract infections (RESP), surgical site infections (SSI) and other infections (OTH). MRSA, methicillin-resistant *Staphylococcus aureus*.

MRSA incidence of infection varied widely among 29 countries across Europe, ranging from an estimated 1.47 cases in the Netherlands to 102 cases in Portugal per 100 000 persons per year (Fig. 4, Figs. S7 and S8). Similarly, antimicrobial usage is markedly different across countries (Figs. S9–S12). We compared antimicrobial usage with MRSA incidence across European countries to further explore MRSA selection by antibiotics.

**Fig. 5:**
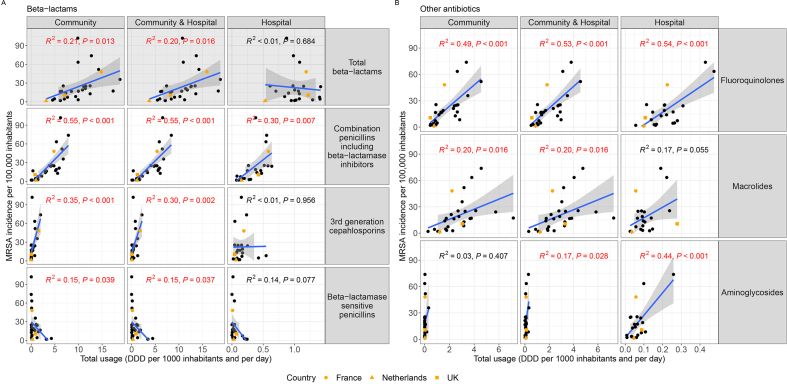
Antimicrobial usage in 29 European countries is associated with MRSA infection incidence. (A) β-lactam (ATC code classes: ‘J01C’ and ‘J01D’) usage in the community, hospitals or combined. (B) For other antibiotics, associations were seen with fluoroquinolones (‘J01MA’), Macrolides (‘J01FA’) and aminoglycosides (‘J01G’) (here the outlier of Portugal was excluded). See Supplementary 2 for all antibiotics. Significant trends are highlighted with a red R^2^ and p value (p < 0.05). Shaded cells indicate summary classes of antibiotics—those that are sums of other columns (Table S1). Shaded areas around the blue trend line are the 95% confidence level interval for predictions from a linear model. MRSA, methicillin-resistant *Staphylococcus aureus*.

MRSA incidence correlated with total β-lactam usage in 29 countries (Fig. 5(a), Figs. S16–S19). Specific associations were found between combinations of penicillins, including β-lactamase inhibitors in the community and hospitals, and third-generation cephalosporins in the community. Countries with higher use of β-lactamase-sensitive penicillins in the community had lower MRSA incidence.

We also identified a correlation between MRSA incidence and fluoroquinolone usage (Figs. 5(b) and S20–S23). We note that Portugal is an outlier in our data with very high MRSA incidence. When excluding Portugal from the analysis, additional correlations were found between MRSA incidence with macrolide use in the community and aminoglycoside use in hospitals (Figs. 5(b), Figs. S24–S27 and S31–S32). Correlations between MRSA incidence and other classes of antimicrobials were not significant (Supplementary Data pages 14–55).

## Discussion

In this project, we brought together isolates from three European countries with varying MRSA incidences. Surprisingly, we found that harmonizing a definition and unifying data analysis for epidemic success was extremely challenging. We explored the internationally differing MRSA surveillance programmes, strain collections, typing methods and uses of data [8]. THD analysis confirmed that our operational definitions of success in each country did not completely match or capture epidemic success. A unified framework for MRSA sampling is needed to establish cohesive sample and data collection, such as common reasons for collecting isolates and sources of isolates, harmonized typing and accessible strains, which we discuss further in an accompanying paper [8]. This would allow a direct comparison between clonal types of differing incidence and success in different locations.

The high variation in MRSA incidence and CC types in European countries indicates differing selection pressures based on geographical factors. Identifying markers of selection in successful isolates in different countries will be key to designing effective interventions to reduce selection.

Comprehensive phylogenetic analysis by GWAS did not identify mutations associated with success. Despite using pyseer, the complex lineage structure of MRSA may have confounded the analysis. This method does not include most of the antibiotic resistances that in MRSA are because of resistance genes carried on mobile genetic elements. A THD analysis was more nuanced assigning indices for success. This approach allowed CC22 to be clearly identified as the most successful clone, despite samples being chosen across a range of CCs.

Across the collection, an antibiotic resistance phenotype was associated with THD success for ciprofloxacin, macrolides and mupirocin (Fig. 3). Success was not associated with gentamicin, rifampicin and trimethoprim resistance. This pattern does not simply align with CC22 AMR profiles, or with resistance profiles in the UK MRSA, and demonstrates that CCs across the strain collection and all three countries contributed to this finding.

If certain AMRs were particularly associated with success, we might expect the antimicrobials to be used at higher frequency in areas where these resistances have become prevalent. We used standardized data and estimates across 29 countries of Europe to answer this question. First, we demonstrated that β-lactam use was correlated with MRSA incidence, and specifically, this could be narrowed down to penicillins combined with β-lactam inhibitors in both hospitals and the community. In addition, there was a correlation with third-generation cephalosporin use in the community. Importantly, higher usage of β-lactamase-sensitive penicillins in the community was correlated with a lower MRSA incidence, likely because of their effectiveness when MRSA incidence is low. These results may provide suggestions as to which β-lactams could be targeted by stewardship interventions and in which locations.

Beyond β-lactams, fluoroquinolone use (including ciprofloxacin) correlated with MRSA incidence across Europe. Resistance to fluoroquinolones because of stable point mutations is common in successful clones, and reduction of ciprofloxacin usage has previously been implicated in MRSA incidence decline in UK hospitals [9], whereas resistance was identified as a key selected epidemiological marker using phylogenetic methods [17,21]. Fluoroquinolone antimicrobials are particularly secreted onto the skin and mucous membranes [22], influencing the colonizing microbiome, and presumably selecting a host reservoir of MRSA.

Macrolide resistance was also implicated as having an association with successful MRSA, despite the instability of the resistance gene in MRSA populations [9,21,23]. Genes are typically carried on plasmids and transposons with a high incidence of gain and loss in experimental and phylogenetic studies [23]. High frequency of resistance in MRSA suggests active selection. Usage of macrolides in the community correlated with MRSA incidence in Europe and further studies should focus on the proportion of resistant MRSA that may vary across countries.

Although mupirocin resistance was identified as a marker of success, the incidence is relatively low, and there is limited previous evidence for mupirocin resistance contributing to epidemiological success. Similarly, *tst* gene (encoding for the toxic shock syndrome toxin) carriage was implicated in success, but the incidence was also low [24]. Aminoglycoside resistance was less prevalent in successful MRSA, although use in hospitals was associated with MRSA incidence, and we can speculate that the large plasmids carrying such resistances may be a burden to colonizing strains. Rifampicin resistance mutations are also rare, possibly because of fitness cost [25].

MRSA isolates showed evidence of recent spread from one country to another, but limited spread in the new location. However, the study was hampered by under-sampling to evidence this. Our recent mathematical modelling has suggested that dominant local clones have a particular advantage in outcompeting introduced clones, particularly when AMR genes are unstable [10].

All epidemiological studies are limited and biased by the isolates chosen to study. Here, we attempted to power our study by selecting successful versus unsuccessful/sporadic isolates, which was hampered by un-harmonized collections. However, the very different strain collections from three different countries hampered our analysis [8]. The choice of isolates may have skewed our THD analysis. THD is a method that benefits from large strain collections, and future studies could investigate global populations assigning success and utilizing comparisons with genotypic AMR.

This study highlights the wide variation in AMR incidence in MRSA populations in the France, the UK and the Netherlands, as well as in the usage of antimicrobials. Furthermore, there are alignments between the use of particular antimicrobial classes and MRSA infection incidence across Europe. Stewardship programmes to reduce infection incidence can be hampered if they focus on restricting antimicrobial usage that is not selective. The data presented here may allow targeted interventions, particularly in locations where MRSA is prevalent. Further studies to investigate the antibiotic resistance profiles of MRSA in a wider range of locations, combined with the impact of changing antimicrobial usage over time, will support the design of enhanced stewardship interventions.

## Author contributions

Conceptualization and supervision: MCV, GMK, AAW, J-PR, JAL. Funding: MCV, GL, JAL. Data generation and analysis: VOB, AG, GMK, LMS, KL, MT, APAH, MK, MCV, AAW, JPR, JAL. Methodology of strain collection: VOB, AG, GMK, LMS, AB, WJBvW, GL, FV, MCV, AAW, J-PR, JAL. Strain collection: VOB, AG, LMS, APAH, MT, J-PR, JAL. Sequencing and bioinformatics: AAW, KL, AG, J-PR. Phenotypic antimicrobial resistance analysis: AG, THD, J-PR. Antibiotic usage analysis: GMK, AG, JAL. MACOTRA consortium member intellectual input: VB, AG, GMK, LMS, AB, SJdV, ASdV, APAH, MEK, WJBvW, GL, FV, MCV, AAW, J-PR, JAL. Writing: VOB, AG, GMK, LMS, MCV, AAW, J-PR, JAL. These authors jointly supervised this work: Adam A. Witney, Jean-Philippe Rasigade, Jodi A. Lindsay. All authors reviewed and agreed with the final version of the manuscript.

## Transparency declaration

The authors declare that they have no conflicts of interest.

## Funding

The MACOTRA (Combating MRSA; increasing our understanding of transmission success will lead to better control of MRSA) study was supported by JPIAMR 3**rd** call AMR Transmission dynamics, UK Medical Research Council grant MR/P028322/1, French ANR Grant 16-JPEC-000 and Dutch ZonMw grant 547001006.
