# Supplementary material for: Markers of epidemiological success of methicillin-resistant Staphylococcus aureus isolates in European populations
Source: Clin Microbiol Infect. 2023 Sep;29(9):1166–73. doi: 10.1016/j.cmi.2023.05.015 (PMC10775016; doi:10.1016/j.cmi.2023.05.015)
Supplement: Multimedia component 1 [file mmc1.docx]

**Supplementary information –**

Methods page 1

Operational definition of epidemiologically successful and sporadic isolates

Strain collection page 3

MRSA resistance phenotype distribution by country and operational success page 10

MRSA infection incidence and Antibiotic Use page 14

References page 56

**METHODS**

***Operational definition and collection of successful and sporadic (unsuccessful) isolates***

Collaborators from each country, France, the Netherlands and the UK, were tasked to identify their country-specific epidemiological characteristics of success over time, which included incidence of infection and identification of dominant clones using local methodology. This approach relied on published and reference centre resources, as well as investigation of local available strain collections using local typing methods. Using this information, the most useful method of identifying successful and sporadic (unsuccessful) isolates within collections was identified and described, and the criteria for identifying individual examples for strain analysis proposed. Where common criteria across countries could be identified this was used.

***Whole genome sequencing and epidemiological clustering***

Whole genome sequencing of isolates was generated at St. George’s University of London, UK (SGUL) using the Illumina MiSeq platform. UK isolates from 2003, 2006, 2008 and 2009 (n= 168) had been previously sequenced by Kime et al. [1]. DNA was extracted from the remaining isolates using the PurElute (Edge Biosystems) kit and 2.5ul of lysostaphin (Sigma Aldrich). Sequence reads were aligned to reference genomes (RefSeq NC_002952, NC_017763, NC_002745) using bwa mem 0.7.17-r1188 and sites called with bcftools mpileup (v1.9) [2]. Sites were filtered based on the following criteria: mapping quality (MQ) above 30, site quality score (QUAL) above 30, at least 4 reads covering each site with at least 2 reads mapping to each strand, at least 75% of reads supporting site (DP4) Sites which failed these criteria in any isolate were removed from the analysis. Phylogenetic reconstruction was performed using RAxML v8.2.3 with a GTR model of nucleotide substitution and a GAMMA model of rate heterogeneity, branch support values were determined using 1000 bootstrap replicates [3]. Genomes were also assembled using Shovill v1.0.9 and resistance genes identified using Abricate and the CARD database, virulence genes identified using the VFDB database [4,5].

***Genome-based estimation of epidemic success***

Assembled genomes were annotated using prokka [6] and the pangenome assessed using roary [7] using default parameters. Genes associated with success and resistance phenotypes were determined using pyseer [8]. First unitigs were counted with unitig-counter [9] and unitigs with significant associations with the phenotypes determined using a linear mixed model, correcting for population structure using a kinship matrix generated by an all isolate phylogenetic tree, generated as described above.

***Antimicrobial susceptibility testing***

EUCAST disk diffusion methodology [10] was used to test for sensitivity to 14 antibiotic disks (Oxoid, Basingstoke, UK): cefoxitin (30 µg), ampicillin (2 µg), chloramphenicol (10 µg), ciprofloxacin (5 µg), clindamycin (2 µg), erythromycin (15 µg), gentamicin (10 µg), tobramycin (10 µg), fusidic acid (10 µg), linezolid (10 µg), mupirocin (200 µg), rifampicin (5 µg), tetracycline (30 µg), and trimethoprim (5 µg). Ninety-five of the UK isolates were previously tested using BSAC criteria [1,27]. Following determination of antimicrobial susceptibility of the MRSA isolate collection, the distribution of phenotypic resistances and successful isolates in each country was compared using Chi-squared tests, p<0.05; if any expected values were < 5, Fisher’s exact test was used.

**Time-scaled haplodensity (THD) analysis**

Phyloepidemiology methods leverage genome sequences to derive epidemiological quantities including epidemic success. We used the time-scaled haplotypic density method to examine the factors predicting epidemic success of MRSA [11–13]. THD assigns relative indices of epidemic success, over a defined time period, to each isolate in the dataset, on the basis of the branching density and distribution of genetic distances separating it from other isolates.

Genetic distances were defined as the pairwise number of SNPs between isolates, based on sequence alignment with respect to the NC017763 MRSA reference genome. THD was computed from the matrix of pairwise SNP distances, using an effective genome size of 3x10^6^ bp and an evolutionary rate of 10^-6^ substitution per site per year as previously determined for *S. aureus* [21]. An epidemic period of 5 years was used as the THD timescale to restrict analysis to short-term success. Four isolates (MAC209, 226, 204, and 188) exhibited THD indices <1e-8 and were excluded. THD analyses were also restricted to isolates belonging to CCs with a sample size >10, namely CC1, 22, 30, 45, 5, 8, 80 and 398, to avoid including outliers in the models. N=368 isolates were included in the final THD analyses.

***Detection of predictors of epidemic success***

Potential predictors of success, such as antimicrobial drug resistance patterns, were identified using linear regression models with THD indices as the response variable. Adjustment for potential confounders was conducted, where indicated in text, by including confounders as model co-variates or as random effects, as appropriate. Associations of predictors with THD indices are reported as regression slope coefficients with 95% confidence intervals computed using likelihood profiling. All analyses used R software version 4.0.2 with additional packages *thd* (<https://github.com/rasigadelab/thd>) and *lmerTest* [14–16].

***AMR resistance and antimicrobial usage***

The sum of the incidence of all infection types caused by MRSA estimated in Cassini *et al*. was used to give an estimate of MRSA annual incidence per 100 000 population data across 29 European countries [17]. This is based on EARS-Net data adjusted for coverage and usage of diagnostics for the year 2015. Antimicrobial consumption data was from ESAC-Net (ecdc.europa.eu) with a focus on the year 2015 and expressed at DDD per 1000 inhabitants per day. The data for some countries is split into community and hospital usage, and is sourced from national sales and reimbursement data. Data manipulation and exploration is presented in the Supplementary with supporting code (ref: <https://github.com/gwenknight/mrsa_inf_abx> for code analysis). Regression models were fitting using the “lm()” function in *R* [15].

**RESULTS**

**Operational definition of epidemiologically successful and sporadic isolates**

**Details of isolate selection**

Country-specific strategies were adopted with different inclusion criteria for storage and surveillance. Detailed information for each strategy is given below.

**France*.*** Representative isolates from France were selected from the collection of the French *National* Reference Centre for Staphylococcus (NRC), Lyon, France. This collection consists of methicillin-susceptible *Staphylococcus aureus* (MSSA) and MRSA isolates referred to the NRC by approximately 380 laboratories for microbiological expertise and does not include isolates from clinical studies or cohorts. From 2014 onwards, all isolates in the collection have been subjected to DNA array profiling using 332-loci Alere Staphytype (Alere Technologies GmbH, Jena, Germany) as described elsewhere [18]. Isolates are assigned to multilocus sequence types (STs) and clonal complexes (CCs), as well as specific lineages such as ST8 USA300 [19], by comparing whole-array hybridization profiles to previously MLST-typed reference strains in a dedicated database [20].

Isolates with a ST8 USA300 profile were readily classified as sporadic in France based on their limited local spread compared to other countries, in spite of repeated introductions [21]. Other isolates were classified as successful or sporadic and stratified across major CCs using the following rationale. Major CCs were defined as those with >20 MRSA isolates in the 2014-17 collection (which had a total of 5,457 isolates including 1,382 MRSA). Eight major CCs were found, namely CC8 (*n = 216*), CC5 (*n = 200*), CC80 (*n = 171*), CC30 (*n =39*), CC22 (*n = 36*), CC1 (*n= 33*), CC88 (*n = 27*) and CC59 (*n = 25*), totalling 747 isolates.

The successful or sporadic classification of isolates was then based on their subtype cluster frequency within each major CC. This rationale for clustering was to ensure that the intra-cluster variability within a CC was constant. First, microarray data were subjected to hierarchical clustering using Ward’s method to produce one dendrogram per CC [22]. Clusters of isolate subtypes were found in each dendrogram using equal-height tree cutting, where the number of clusters was arbitrarily defined as one-fifth of the number of isolates in the CC, up to a maximum of 10 clusters. This method ensured consistent subtyping of isolates across CCs of varying size. Subtype clusters in each CC were sorted by size. The largest clusters totalling >25% of CC size were labelled as ‘successful’ while the smallest clusters totalling >25% of CC size were labelled as ‘sporadic’. Other subtype clusters were considered inconclusive and excluded.

The above classification criteria resulted in 316 isolates classified as ‘successful’ (*CC8 = 97, CC5 = 81, CC80 = 67, CC30 = 21, CC22 = 11, CC1 = 13, CC88 = 18 and CC59 = 8*) and 152 isolates as ‘sporadic’ (*CC8 = 40, CC5 = 37, CC80 = 42, CC30 = 9, CC22 = 8, CC1 = 4, CC88 = 6 and CC59 = 6*). A final subset of 96 isolates were selected using balanced sampling across CCs, as well as between successful and sporadic isolates within each CC.

**Netherlands. *Type-Ned MRSA database*** - As surveillance and collection of MRSA isolates along with relevant epidemiological data is mandatory in the Netherlands, the Dutch National Institute for Public Health and the Environment (Rijksinstituut voor volksgezondheid en milieu [RIVM]) has been receiving and storing MRSA isolates collected through the national surveillance system. This system includes all Dutch Medical Microbiological Laboratories (MML) associated with general practitioners, regional and university hospitals, long term care facilities, and laboratories in Dutch territories overseas. One isolate per person per year is included. These include clinical isolates as well as colonisation isolates, irrespective of the reason for detection, either by contact search, increased risk factors (see below) or clinical samples. When both colonisation and clinical isolates are available, a clinical isolate is preferred, but in practice the first isolated MRSA from a person will be included. All data is collected in the Type-Ned MRSA database. This includes MML of submission, all relevant personal data and epidemiological data, such as gender, age and sample site. Patient privacy is guaranteed under the Dutch law.

Following search and destroy (S&D), a policy implemented in the Netherlands since 1988, every patient at risk for MRSA colonisation is screened at hospital or nursing home admission and placed in pre-emptive isolation awaiting culture results. Subsequently, patients with MRSA positive culture are kept in strict isolation during their hospital stay and offered a treatment to eliminate colonisation, preferably and mostly after discharge and being recovered. Before treatment, household members are tested on carriage and thus transmission and offered an elimination treatment together and at the same moment with the index carrier when positive. Risks for MRSA colonisation were defined by the former Dutch Working party for Infection Prevention (WIP; 1981-2017) and include, among others, contact with an MRSA carrier, recent stay in a hospital abroad and contact with farmed pigs, veal calves or broilers [23]. The assumed origin of MRSA acquisition is classified by infection control practitioners, based on the WIP risk categories, and reported in the Type-Ned database. Occasionally, MRSA is isolated from patients not targeted by S&D, for example in a clinical sample (MRSA of unknown origin; MUO [24,25]. These findings result in contact tracing which aims to screen all exposed contacts to detect and prevent MRSA outbreaks. Sometimes, this results in identifying a MRSA isolate of different genetic origin than the original MRSA isolate for which the contact search was initiated. These isolates are defined as unexpected findings which start new contact tracings. When no transmission of these unexpected MRSA types is found and their prevalence in the Netherlands is low, we define these MRSA types as unsuccessful (sporadic), as these were carried by hospitalised patients without any contact precautions, and did not show transmission , where another MRSA type in that particular hospital setting had spread. These in particular identified isolates were included as unsuccessful isolates NL4 (see below).

***Strain selection***

The period 2008-2017 was chosen to ensure overlap in time with the selection period of British and French isolates. During 2008-2017, ±32.000 MRSA isolates were collected through national surveillance. Aside from livestock-associated (LA) MRSA clade MC0398, the following MLVA -Complexes (MCs) were most prevalent: MC0005, MC0008, MC0022, MC0045, MC0030 and MC0001 (Table S1). As the latter six MC corresponded with frequently found MLST-CC in the UK and France, a subset of isolates belonging to these MC were selected for MACOTRA. To narrow our search and account for changes in prevalence over time, we chose to select isolates from sampling years 2008 and 2017 only. We aimed to select 12 isolates per all six MCs consisting of six isolates for each sampling year per MC. During selection, isolates originating from as many different MMLs as possible were chosen. If a further choice was possible, the earliest submitted isolates were preferred. Four independent selection methods (described below and depicted in Figure S6) were used to complete the collection Dutch isolates.

|  | Prevalence per sampling year | | | | | | | | | |  |
| --- | --- | --- | --- | --- | --- | --- | --- | --- | --- | --- | --- |
| MLVA-Complex | 2008 | 2009 | 2010 | 2011 | 2012 | 2013 | 2014 | 2015 | 2016 | 2017 | Total |
| MC0398 | 41 | 42 | 40 | 40 | 38 | 34 | 30 | 28 | 25 | 25 | 34 |
| MC0005 | 15 | 14 | 15 | 15 | 15 | 14 | 18 | 13 | 16 | 13 | **15** |
| MC0008 | 15 | 16 | 14 | 14 | 13 | 16 | 14 | 12 | 12 | 12 | **14** |
| MC0022 | 5 | 5 | 8 | 5 | 6 | 7 | 8 | 12 | 9 | 12 | **8** |
| MC0045 | 8 | 6 | 5 | 8 | 8 | 8 | 11 | 10 | 7 | 6 | **8** |
| MC0030 | 3 | 4 | 4 | 4 | 5 | 5 | 4 | 5 | 6 | 6 | **5** |
| MC0001 | 1 | 2 | 2 | 1 | 2 | 2 | 3 | 4 | 5 | 5 | **3** |
| Other MCs | 12 | 12 | 12 | 12 | 14 | 12 | 13 | 16 | 20 | 20 | 15 |

Supplementary Table S2. Relative prevalence (%) of included MC in the Netherlands per sampling year

***Selection of successful isolates***

Individual minimum spanning trees (MST) based on MLVA-types were made for MC0005, MC0008, MC0022, MC0030, MC0045 and MC0001. These MCs are representative of CC5, CC8, CC22, CC30, CC45 and CC1, respectively (expert opinion, Leo M. Schouls). Subsequently, the most prevalent MLVA types were chosen within each MC. From these MLVA types, approximately 8 isolates were selected at random and these were categorized as successful MRSA as they have been able to persist and spread throughout the study period. MC0001 was considered least successful of the six selected prevalent MCs, hence, only 4 isolates from the most prevalent MLVA types were included as successful isolates. This selection method was named NL1. As LA-MRSA, MC0398 is the most prevalent MC in the Netherlands, a separate MST of MC0398 was used to expand the set of the above successful isolates. Three isolates from each sampling year for the most prevalent MLVA types within MC0398 were selected. This selection method was defined as NL2.

***Selection of sporadic isolates***

For selecting sporadic isolates, four isolates were selected from rare MLVA types within each MC specific MST used in method NL1. These isolates were categorized as sporadic MRSA. For MC0001, 8 isolates from rare MLVA types were included. The collection was expanded with another six isolates from globally dominant clones, which are not prevalent in the Netherlands. Based on epidemiological data, these isolates were categorized as unsuccessful in the Netherlands, as these clones were unable to cause outbreaks in a hospital setting despite repeated introduction (selection method NL3). The added isolates included a pair of ST239 (MC0008) isolates, a pair of USA300 (defined as PVL+ and *spa* type t008) isolates and a pair of MC0080 isolates, with one isolate from 2008 and another from 2017 for each pair.

The set of sporadic isolates was further expanded with six isolates originating from unexpected findings during contact tracing of MRSA outbreaks in Erasmus MC hospital between 2008 and 2017 (selection method NL4). As described above, these MRSA had the chance to spread in a hospital setting but did not show any transmission i.e. were unsuccessful. Furthermore, the MLVA types of these last 6 isolates were present less than 5 times in the Dutch Type-Ned MRSA database between 2008 and 2017.

***Additional outbreak isolates***

Next-generation sequencing (NGS) of MRSA has been implemented at RIVM since 2017, enabling outbreak investigations based on whole genome MLST (wgMLST). For this approach, 2567 loci of the core and accessory genome were included, and importantly grouping based on wgMLST agreed between NGS groups and MLVA complexes [26]. The average allelic distance between NGS groups was 1673 alleles, ranging between 1169 and 1959 alleles. Genetic clusters representing possible outbreak clusters were defined as isolates within a NGS group separated by a maximum of 15 genes. In total, 20 isolates were included from five different genetic clusters (range 1- 12 alleles). From each selected genetic cluster, two isolates were defined as successful. For each genetic cluster two genetically closely related, but outside of the genetic cluster (range 43-288 alleles) were selected as sporadic counterparts.

In total, 109 isolates were included in the Dutch part of the MACOTRA strain collection.


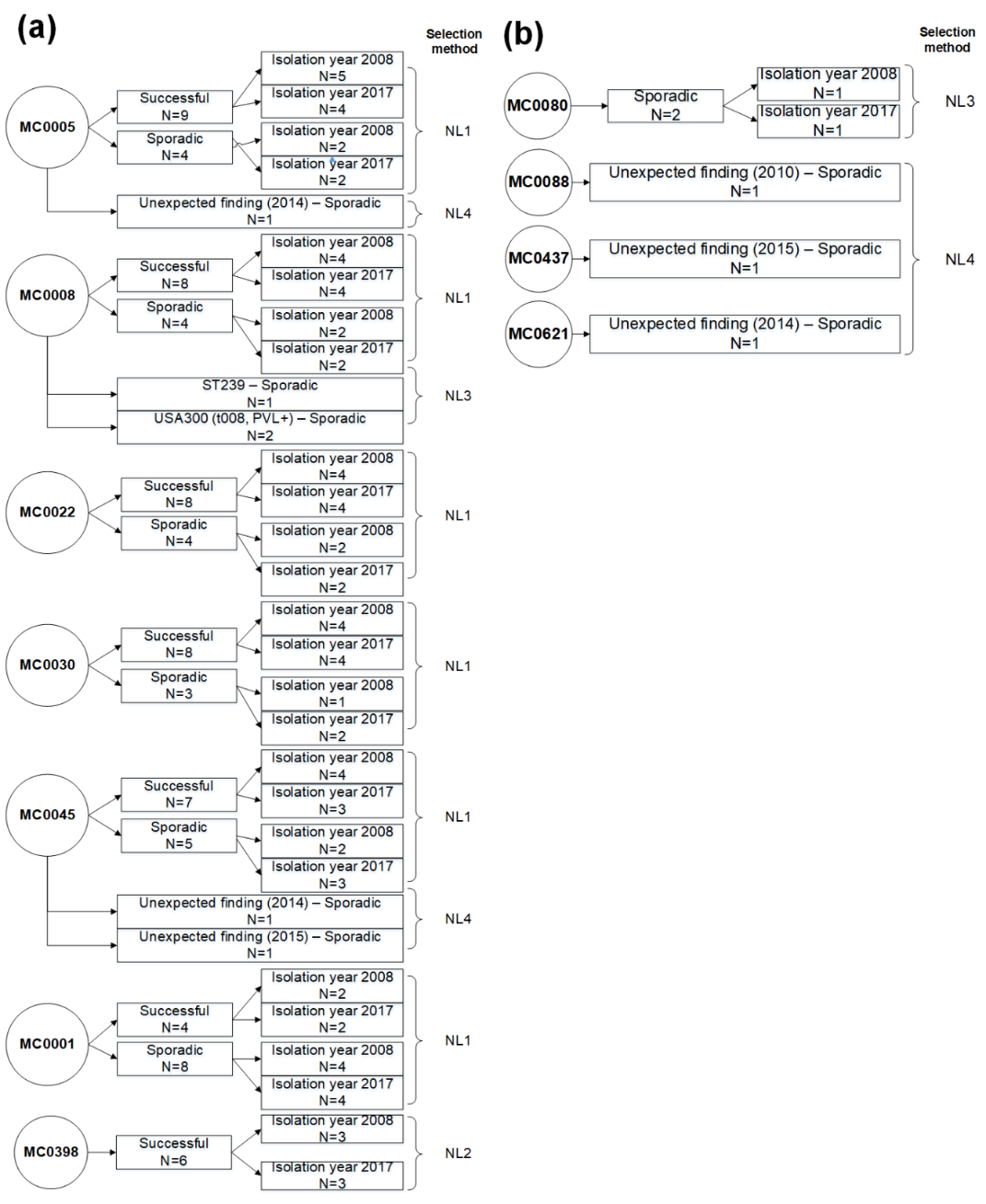


**Figure S6.** **Selection procedure for Dutch isolates.** (a) illustrates the selection procedure of successful and sporadic from the most prevalent MLVA-MC found in the Netherlands; includes LA-MRSA clade, MC0398; (b) describes the selection of sporadic isolates from less prevalent MLVA-MC identified in the Netherlands. NL1: based on selection of prevalent and rare MLVA types of 6 prevalent MLVA-MCs; NL2: selection of prevalent MLVA types of MC0398; NL3: selection based on global successful clones; NL4: selection based on unexpected findings in contact tracings. Unexpected findings implies MRSA of unknown origin, no transmission detected in contact tracing, prevalence <0.025% in MRSA Type-Ned database.

**United Kingdom*:*** For isolates selection, a collection of well characterised isolates from a single London hospital was utilized. These isolates were representative of the region, and were collected both before and after the reduction in incidence of MRSA infection in the UK in 2007 [27]. Since then the dominant clones of MRSA in the UK have remained fairly constant (28). These St George’s University Hospital Trust, London isolates were collected from 1999 – 2009 from a range of specimens sent to the diagnostic microbiology laboratory in a large acute teaching hospital servicing south-west London. In 1999 and 2003, the dominant clone was CC30, interrupted by the emergence and decline of the ST239 clone, and by 2006 were dominated by CC22. Most isolates were resistant to ciprofloxacin and erythromycin. Additionally, resistance to aminoglycoside, trimethoprim, fusidic acid and tetracycline were seen. All isolates had been subjected to WGS [1] and lineages were confirmed. The collection was supplemented with all stored blood culture MRSA isolates collected at St George’s between 2013-2016, where CC22 remained the dominant clone.

For the total 173 isolates, all those belonging to CC1, CC5, CC8, CC45, ST239, CC51, CC59 (n=29, 16.8% of the collection) were classified as sporadic owing to their relatively rare occurrence. For CC22 and CC30 isolates, phylogenetic trees of the collections from all three countries were constructed (Figures S1 and S2 in Supplementary Figures file), and we defined 'successful' as those UK isolates that belonged to a cluster of two or more isolates on the tree with a SNP difference of <15 bp. The collection assigned 61 successful and 112 sporadic isolates, only 82 isolates were used in the final analysis.

**MRSA resistance phenotype distribution by country and operational success**

**Supplementary Table S3**

| Results from Chi2/Fisher's exact test in R | | | | |  |  |  |  |  |  |  |
| --- | --- | --- | --- | --- | --- | --- | --- | --- | --- | --- | --- |
|  | |  |  |  |  |  |  |  |  |  |  |
|  |  |  |  |  |  |  |  |  |  |  |  |
|  |  |  |  |  |  |  |  |  |  |  |  |
|  |  |  |  |  |  |  |  |  |  |  |  |
|  |  |  |  |  |  |  |  |  |  |  |  |
|  | |  |  |  |  |  |  |  |  |  |  |
| antibiotic | | country | R | S | Total | perc | Test | X | df | p |  |
| ciprofloxacin | | FR | 44 | 52 | 96 | 0.46 | chi2 | 94.76 | 2 | < 2.2e-16 |  |
|  | | NL | 44 | 65 | 109 | 0.40 |  |  |  |  |  |
|  | | **UK** | 157 | 16 | 173 | 0.91 |  |  |  |  |  |
|  | |  |  |  |  |  |  |  |  |  |  |
| antibiotic | | country | R | S | Total | perc | Test | X | df | p |  |
| erythromycin | | FR | 27 | 69 | 96 | 0.28 | chi2 | 74.28 | 2 | < 2.2e-16 |  |
|  | | NL | 46 | 63 | 109 | 0.42 |  |  |  |  |  |
|  | | **UK** | 136 | 37 | 173 | 0.79 |  |  |  |  |  |
|  | |  |  |  |  |  |  |  |  |  |  |
| antibiotic | | country | R | S | Total | perc | Test | X | df | p |  |
| fusidic acid | | **FR** | 27 | 69 | 96 | 0.28 | chi2 | 14.47 | 2 | 0.00072 |  |
|  | | NL | 13 | 96 | 109 | 0.12 |  |  |  |  |  |
|  | | UK | 20 | 153 | 173 | 0.12 |  |  |  |  |  |
|  | |  |  |  |  |  |  |  |  |  |  |
| antibiotic | | country | R | S | Total | perc | Test | X | df | p |  |
| gentamicin | | FR | 6 | 90 | 96 | 0.06 | chi2 | 16.7 | 2 | 0.000233 |  |
|  | | NL | 12 | 97 | 109 | 0.11 |  |  |  |  |  |
|  | | **UK** | 41 | 132 | 173 | 0.24 |  |  |  |  |  |
|  | |  |  |  |  |  |  |  |  |  |  |
| antibiotic | | country | R | S | Total | perc | Test | X | df | p |  |
| mupirocin | | FR | 0 | 96 | 96 | 0.00 | chi2 | 11.3 | 2 | 0.003516 |  |
|  | | NL | 5 | 104 | 109 | 0.05 |  |  |  |  |  |
|  | | UK | 17 | 156 | 173 | 0.10 |  |  |  |  |  |
|  | |  |  |  |  |  |  |  |  |  |  |
| antibiotic | | country | R | S | Total | perc | Test | X | df | p |  |
| tetracyclin | | FR | 17 | 79 | 96 | 0.18 | chi2 | 16.47 | 2 | 0.000265 |  |
|  | | **NL** | 32 | 77 | 109 | 0.29 |  |  |  |  |  |
|  | | UK | 17 | 155 | 172 | 0.10 |  |  |  |  |  |
|  | |  |  |  |  |  |  |  |  |  |  |
| antibiotic | | country | R | S | Total | perc | Test | X | df | p |  |
| tobramycin | | FR | 23 | 73 | 96 | 0.24 | chi2 | 9.66 | 2 | 0.007972 |  |
|  | | **NL** | 18 | 91 | 109 | 0.17 |  |  |  |  |  |
|  | | **UK** | 57 | 116 | 173 | 0.33 |  |  |  |  |  |
|  | |  |  |  |  |  |  |  |  |  |  |
| antibiotic | | country | R | S | Total | perc | Test | X | df | p |  |
| trimethoprim | | **FR** | 7 | 89 | 96 | 0.07 | chi2 | 17.44 | 2 | 0.000163 |  |
|  | | NL | 15 | 94 | 109 | 0.14 |  |  |  |  |  |
|  | | UK | 46 | 127 | 173 | 0.27 |  |  |  |  |  |
|  | |  |  |  |  |  |  |  |  |  |  |
|  | |  |  |  |  |  |  |  |  |  |  |
|  | |  |  |  |  |  |  |  |  |  |  |

**Supplementary Table S4**

| Results from Chi2/Fisher's exact test in R | | | |  |  |  |  |  |  |  |  |
| --- | --- | --- | --- | --- | --- | --- | --- | --- | --- | --- | --- |
| **Overall** |  |  |  |  |  |  |  |  |  |  |  |
| antibiotic | success | R | S | Total | perc | Test | X | df | p |  |  |
| tobramycin | unsuccessful | 48 | 173 | 221 | 0.22 | chi2 | 4.3893 | 1 | 0.03616 |  |  |
|  | **successful** | 50 | 107 | 157 | 0.32 |  |  |  |  |  |  |
|  |  |  |  |  |  |  |  |  |  |  |  |
| **France** |  |  |  |  |  |  |  |  |  |  |  |
| antibiotic | success | R | S | Total | perc | Test | p | 95CI_min | 95CI_max | OR |  |
| clindamycin | **unsuccessful** | 10 | 54 | 64 | 0.16 | fisher's | 0.03 | 1.22 | Inf | Inf |  |
|  | successful | 0 | 32 | 32 | 0.00 |  |  |  |  |  |  |
|  |  |  |  |  |  |  |  |  |  |  |  |
| antibiotic | success | R | S | Total | perc | Test | X | df | p |  |  |
| tetracycline | **unsuccessful** | 16 | 48 | 64 | 0.25 | chi2 | 5.58 | 1 | 0.01812 |  |  |
|  | successful | 1 | 31 | 32 | 0.03 |  |  |  |  |  |  |
|  |  |  |  |  |  |  |  |  |  |  |  |
| antibiotic | success | R | S | Total | perc | Test | X | df | p |  |  |
| tobramycin | unsuccessful | 10 | 54 | 64 | 0.16 | chi2 | 6.01 | 1 | 0.01422 |  |  |
|  | **successful** | 13 | 19 | 32 | 0.41 |  |  |  |  |  |  |
|  |  |  |  |  |  |  |  |  |  |  |  |
| **UK** |  |  |  |  |  |  |  |  |  |  |  |
| antibiotic | success | R | S | Total | perc | Test | X | df | p |  |  |
| ciprofloxacin | unsuccessful | 96 | 16 | 112 | 0.86 | chi2 | 7.98 | 1 | 0.004741 |  |  |
|  | **successful** | 61 | 0 | 61 | 1.00 |  |  |  |  |  |  |
|  |  |  |  |  |  |  |  |  |  |  |  |
| antibiotic | success | R | S | Total | perc | Test | X | df | p |  |  |
| gentamicin | unsuccessful | 17 | 95 | 112 | 0.15 | chi2 | 11.45 | 1 | 0.000714 |  |  |
|  | **successful** | 24 | 37 | 61 | 0.39 |  |  |  |  |  |  |
|  |  |  |  |  |  |  |  |  |  |  |  |
| antibiotic | success | R | S | Total | perc | Test | p | 95CI_min | 95CI_max | OR |  |
| rifampicin | unsuccessful | 0 | 112 | 112 | 0.00 | fisher's | 0.01 | 0 | 0.803 | 0 |  |
|  | **successful** | 4 | 57 | 61 | 0.07 |  |  |  |  |  |  |
|  |  |  |  |  |  |  |  |  |  |  |  |
| antibiotic | success | R | S | Total | perc | Test | X | df | p |  |  |
| tetracycline | **unsuccessful** | 18 | 94 | 112 | 0.16 | chi2 | 9.29 | 1 | 0.002309 |  |  |
|  | successful | 0 | 61 | 61 | 0.00 |  |  |  |  |  |  |
|  |  |  |  |  |  |  |  |  |  |  |  |
| antibiotic | success | R | S | Total | perc | Test | X | df | p |  |  |
| tobramycin | unsuccessful | 30 | 82 | 112 | 0.27 | chi2 | 4.7 | 1 | 0.03021 |  |  |
|  | **successful** | 27 | 34 | 61 | 0.44 |  |  |  |  |  |  |
|  |  |  |  |  |  |  |  |  |  |  |  |

**Virulence factors association with success.**

We used the Virulence Factor Database (VFDB) and Abricate to identify the presence or absence of known virulence genes in the whole genome sequences of the strain collection (Table 1). The method has limitations and carriage of a variant gene can be mislabelled as missing. It is also known that many of these genes vary according to lineage [30, 31]. Since we specifically chose isolates of particular lineages in our study, our study design may not support a conclusion that these lineage specific genes were associated with success. In contrast, if genes are carried on mobile genetic elements (MGEs) that are known to move between isolates at high frequency, our study design is suitable for identifying associations with success. MGEs in *S. aureus* encode virulence genes on plasmids, bacteriophage, *S. aureus* pathogenicity islands, and antimicrobial resistance genes on plasmids, transposons and SCC*mec* [32].

Using the operational definition of success, we found an association with toxic shock syndrome toxin gene (*tst)*, which is known to be carried on an MGE *S. aureus* pathogenicity island. However the incidence of *tst* was low in the collection, and therefore the evidence for a role in success is limited.

Using the THD definition of success, we found an association with *lukF-PV* but not *lukS-PV*. These two components encode the full Panton Valentin leukocidin, and the genes are known to be carried on an MGE bacteriophage. *lukF-PV* and *lukS-PV* are very closely homologous to other two-component leucokidin genes in *S. aureus*, and the Abricate method is not conclusive in differentiating between the leukocidin genes. *lukF-PV* was predominantly found in lineages CC8 and CC80 and was more prevalent in France. Overall, we did not conclude that presence of *lukF-PV* in this collection was associated with success, and further work to prove an association would be required.

All data is presented in Table 1.

**MRSA infection incidence and Antibiotic Use**

**Code**

All data and code to recreate the below analysis is provided as code within the Github repository: <https://github.com/gwenknight/mrsa_inf_abx>. All analysis was done in *R* using the “geom_smooth” smooth function within the tidyverse package to visual trend lines and the “ggpmisc” package for the statistical analysis.

**MRSA infection incidence**

We extracted the data from the country specific results in the appendix of Cassini et al (2018) for MRSA to determine the total infection incidence due to MRSA in 2015 (Figure S7). This was a sum across all the infection types estimated (models 61 – 65 in the original Cassini paper notation). There was substantial variation in the total incidence across the 30 European countries, with no estimates available for Iceland (Figure S7A, Figure S8). The proportion of infection incidence due to each infection type was relatively constant (linked to the estimating methods) (Figure S7B). We used the median total infection incidence for the main analysis.

Figure S7: (A) Median infection incidence due to MRSA in 2015 as estimated by Cassini et al. for five infection types (colours) and (B) the relative proportions across these infection types in alphabetical country order. The five infection types are: bloodstream infections (BSI), urinary tract infections (UTI), respiratory tract infections (RESP), surgical site infections (SSI), and other infections (OTH).

Figure S8: Total incidence with 97.5-102.5% confidence ranges from Cassini et al 2018 per country

**Antibiotic use**

Data on antibiotic use was extracted from the ECDC database: <https://www.ecdc.europa.eu/en/antimicrobial-consumption/surveillance-and-disease-data/database> generated from ESAC-Net data submitted to TESSy. All groupings were extracted: such as high-level usage (e.g. antibacterial for systemic and the combination penicillins with beta-lactamase inhibitors), sub-groupings (e.g. 1^st^ generation quinolones) and individual ATC code antibiotics (e.g. tetracyclines at J01A).

Antibiotic use was split into usage in the community and hospital sector. Here we explore the association with (1) community, (2) hospital and (3) combined community and hospital use.

**Codes**

All antibiotics were referred to by their Anatomical Therapeutic Chemical (ATC) code from the World Health Organization where they exist (https://www.whocc.no/atc_ddd_index/?code=j01). These unique codes are assigned to all medicines according to the organ or system it works on and how it works. See Table S5 for a full list of the ATC codes, sub groupings and new additional codes assigned to these sub-groupings for this analysis. These additional codes were introduced to aid in simple visualisation of the data and represent classes given in the data - for example for the different quinolone generations and different durations of macrolide actions (Table S5). Summary antibiotics (in bold in Table S5) are classes of data which represent the sum of sub-groupings of use given in the data.

All antibiotics used here were in the “J01” “Antibacterials for systemic use” classification which does not include antimycobacterials (J04). Within this each antibiotic is grouped J01 with the A-F, M, R and X by mode of action and chemistry. J01R contains two or more systemic antibacterials from different third levels, except combinations of sulphonamides and trimethoprim, which are classified at a separate 4th level, J01EE.

Table S5: Key to ATC code and antibiotic groupings with the hierarchy used in this work. *Additional sub codes are groupings from the data not defined explicitly within the ATC code grouping that were used in this analysis. Those antibiotics in bold represent summary classes of individual or sub-groups of antibiotics (e.g. beta-lactams, or quinolones).

| **ATC code** | **Antibiotic** | **ATC code** | **Antibiotic** | **Sub code*** |  |
| --- | --- | --- | --- | --- | --- |
| J01A | Tetracyclines |  |  |  |  |
| J01B | Amphenicols |  |  |  |  |
| J01C | **Total beta-lactam** | / | All beta-lactams | J01C_D | Sum of J01C and J01D, all beta-lactam |
| J01C | **Beta-lactam antibacterials, penicillins** | J01CA | Penicillins with extended spectrum |  |  |
|  |  | J01CE | Beta-lactamase sensitive penicillins |  |  |
|  |  | J01CF | Beta-lactamase resistant penicillins |  |  |
|  |  | J01CG | Beta-lactamase inhibitors |  |  |
|  |  | J01CR | Combinations of penicillins, incl. beta-lactamase inhibitors |  |  |
| **J01D** | **Other beta-lactam antibacterials** | J01DB | First-generation cephalosporins |  |  |
|  |  | J01DC | Second-generation cephalosporins |  |  |
|  |  | J01DD | Third-generation cephalosporins |  |  |
|  |  | J01DE | Fourth-generation cephalosporins |  |  |
|  |  | J01DF | Monobactams |  |  |
|  |  | J01DH | Carbapenems |  |  |
|  |  | J01DI | Other cephalosporins and penems |  |  |
| **J01E** | **Sulphonamides and trimethoprim** | J01EA | Trimethoprim and derivatives |  |  |
|  |  | J01EB | Short-acting sulphonamides |  |  |
|  |  | J01EC | Intermediate-active sulphonamides |  |  |
|  |  | J01EE | Combinations of sulphonamides and trimethoprim, incl. derivatives |  |  |
| **J01F** | **Macrolides, Lincosamides, Streptogramins** | **J01FA** | **Macrolides** | J01FA_shrt | Short-acting macrolides |
|  |  |  |  | J01FA_intr | Intermediate-acting macrolides |
|  |  |  |  | J01FA_long | Long-acting macrolides |
|  |  | J01FF | Lincosamides |  |  |
|  |  | J01FG | Streptogramins |  |  |
| J01G | Aminoglycoside | J01GB | Other aminoglycosides |  |  |
| **J01M** | **Quinolone** | **J01MA** | **Fluoroquinolones** | J01M_scnd | 2^nd^ generation quinolones |
|  |  |  |  | J01M_thrd | 3^rd^ generation quinolones |
|  |  | J01MB | Other quinolones | J01M_frst | 1^st^ generation quinolones |
| J01X | Other antibacterials | J01XA | Glycopeptide antibacterials |  |  |
|  |  | J01XB | Polymixins |  |  |
|  |  | J01XC | Steroid antibacterials |  |  |
|  |  | J01XD | Imidazole derivatives |  |  |
|  |  | J01XE | Nitrofuran derivatives |  |  |
|  |  | J01XX | Other antibacterials (e.g. Fosfomycin, linezolid) |  |  |

**Variation by country**

The variation by country for 2015 is shown in the Figure S9 (by code) & Figure S10 (by name) below where the highest-level groupings mentioned above have been removed (antibacterial for systemic and the combination penicillins with beta-lactamase inhibitors). The clear greater use of antibiotics in the community can be seen by comparing the x axis ranges for the community (left panels Figure S9 & S10) with those for the hospital (right panels Figure S9 & S10).

Beta-lactam (ATC code J01C) antibiotics are the most commonly used (Figure S11 top), mostly driven by community use of combination penicillins with beta-lactamase inhibitors. For other beta-lactams (ATC code J01D), the most widely used antibiotic were 2^nd^ generation cephalosporins (Figure S11 bottom).

For quinolones, most used are from the fluoroquinolone (J01MA) subgroup (Figure S12, note near complete overlap between light blue dots for fluoroquinolones, pink dots and crosses for total quinolone use).

The types of macrolides were categorized as short- (J01FA01, J01FA02), intermediate- (J01FA06, J01FA09, A02BD04) and long-acting (J01FA10), according to their mean plasma elimination half-life [Adriaenssens](javascript:;) et al (https://academic.oup.com/jac/article/66/suppl_6/vi71/680457). This means that short- is erythromycin and spiramycin, intermediate is roxithromycin, clarithromycin and “pantoprazole, amoxicillin and clarithromycin” with long acting being azithromycin.

Figure S9: Variation in antibiotic use (x axis) and type by ATC Code or sub code (colour) by country (row) and setting (panel column). Note the variation in x axis ranges.

Figure S10: Variation in antibiotic use (x axis) and type by name (colour) by country (row) and setting (panel column). Note the variation in x axis ranges.

Figure S11: Variation in beta-lactam use (x axis) by country (row) and setting (panel column) for Beta-lactams (J01C) and Other Beta-lactams (J01D).

Figure S12: Variation in Quinolone use (x axis) by country (row) and setting (panel column). The crosses (and pink dot) indicate and emphasise total Quinolone use.

**Incidence against antibiotic use**

Overall plots of the trends in antibiotic use in different settings (Community, Hospital and combined) against MRSA infection incidence are shown in the below plots (Figures S13 and S14). Note the variation and also the uncertainty in the linear association trend lines plotted.

Figure S13: Total MRSA infection incidence (y axis) against antibiotic usage (x axis) for each antibiotic grouping (panel) across each setting (colour, red = community, blue = hospital, green = combined community and hospital) with linear trend lines.

Figure S14: Relationship between antibiotic usage (x axis) and infection incidence (y axis) for different antibiotic groupings (rows) and settings in which the antibiotics were given (columns and colours). Trend line is a simple linear association. Note the varying x scales. The plus symbols indicate the 3 countries in the MACOTRA study.

**Exponential vs linear relationship**

Visually (Figure S13), the relationship between antibiotic use and total MRSA infection incidence appeared non-linear for some combinations (e.g. beta-lactam use in the community). We tested this by fitting linear (y~x) and exponential models (log(y)~x) to the data using the “lmList” function in the “nlme” R package [ref: Pinheiro J, Bates D, R Core Team (2022). *nlme: Linear and Nonlinear Mixed Effects Models*. R package version 3.1-160, <https://CRAN.R-project.org/package=nlme>.].

We then compared the R^2^ values of the model fits for those with a *p*-value for the model less than 0.05 i.e. we explored models where there was evidence that there was a significant trend in MRSA infection incidence with changing antibiotic use and asked if an exponential or linear model provided a better explanation of the variance (greater R^2^ value). We found that several antibiotics, examples shown in Figure S15, had a better fit for antibiotic usage and MRSA infection incidence using an exponential model (e.g. 2G_quinolones and beta-lactams).

For simplicity and parsimony, only the linear model outputs are reported in the main paper analysis.

Figure S15: Examples of model fits linking MRSA infection incidence (y axis) with antibiotic usage (x axis) that have non-zero trends and are linear (top, y~x) or exponential (middle/bottom, y~exp(x)). For the latter, a linear model is fit to the logarithmically transformed incidence data (middle row), with the y~exp(x) model output shown in the bottom row. The larger R^2 values are shown in red indicating a better fit.

**Beta-lactam usage and MRSA infection incidence**

When we explored the correlation between beta-lactam usage and MRSA infection incidence, we found a range of significant correlations under a linear (Figure S16) and exponential model (Figure S18). However, the highly strong correlation between hospital beta-lactamase inhibitor use (J01CG) and MRSA infection incidence was tenuous due to its reliance on only two data points: only Portugal and Germany report using any of this J01CG category and only in the hospital. Similarly, the association between carbapenems (J01DH) and MRSA infection incidence is driven by the single datapoint from Portugal (compare below with Figures S16/18 and without Figures S17/19 Portuguese data respectively).

Portugal has a significantly higher MRSA infection incidence (see Figure S7 & S8) and so we explored the usage / incidence correlations with and without the Portugal data. When we removed Portugal from the data, similar trends were seen apart from those with J01CG and J01DH as mentioned above. The association with J01D (“other b-lactams”), which was only significant under an exponential model, also became significant under a linear model without Portuguese data.

*Summary*

For total beta-lactam use (the sum of J01C and J01D, coded here as “J01C_D”), there was a significant trend between community usage and MRSA infection incidence across all models (Figure S16-S19).

For “beta-lactams” (J01C), with or without the data from Portugal, assuming a linear (Figure S16 & S17) or exponential relationship (Figure S18 & S19), there is a significant positive correlation between community use (J01C) and MRSA infection incidence. Exploring the sub-classes of beta-lactams suggests that this is

- due to the positive correlation between all use of combination penicillins including beta-lactamase inhibitors (J01CR) such as ampicillin/amoxicillin/piperacillin and a beta-lactamase inhibitor (ref: <https://www.whocc.no/atc_ddd_index/?code=J01CR&showdescription=no>) under both models and with or without Portuguese data
- and despite the negative correlation between use in the community (linear) and use in the hospital (exponential) of beta-lactamase sensitive penicillins (J01CE) under both models and with or without Portuguese data.

For “other beta-lactams” (J01D), a significant positive correlation between community use and MRSA infection incidence is seen under all analysis except for linear models with Portuguese data. Exploring the sub-classes of “other beta-lactams” suggests that this is driven by significant positive correlations between community use of

- third-generation cephalosporins (J01DD) under both models and with or without Portuguese data
- fourth-generation cephalosporins (J01DE) under a linear model, without Portuguese data
- second-generation cephalosporins (J01DC) under an exponential model with or without Portuguese data

Figure S16: Correlation between antibiotic usage (x axis) and MRSA infection incidence (y axis) for the beta-lactam antibiotic classes assuming a linear relationship. Significant trends are highlighted with a red R^2 and p-value (p<0.05). Shaded cells indicate summary classes of antibiotics – those that are sums of other columns (“All beta-lactams” (J01C_D), “beta-lactams” (J01C), “other beta-lactams” (J01D), see Table S5). Shaded areas around the blue trend line are the 95% confidence level interval for predictions from the linear model.

Figure S17: Correlation between antibiotic usage (x axis) and MRSA infection incidence (y axis) for the beta-lactam antibiotic classes assuming a linear relationship without the data from Portugal. Significant trends are highlighted with a red R^2 and p-value (p<0.05). Shaded cells indicate summary classes of antibiotics – those that are sums of other columns (“All beta-lactams” (J01C_D), “beta-lactams” (J01C), “other beta-lactams” (J01D), see Table S5). Shaded areas around the blue trend line are the 95% confidence level interval for predictions from the linear model.

Figure S18: Correlation between antibiotic usage (x axis) and MRSA infection incidence (y axis) for the beta-lactam antibiotic classes assuming an exponential relationship. Significant trends are highlighted with a red R^2 and p-value (p<0.05). Shaded cells indicate summary classes of antibiotics – those that are sums of other columns (“All beta-lactams” (J01C_D), “beta-lactams” (J01C), “other beta-lactams” (J01D), see Table S5). Shaded areas around the blue trend line are the 95% confidence level interval for predictions from the linear model.

Figure S19: Correlation between antibiotic usage (x axis) and MRSA infection incidence (y axis) for the beta-lactam antibiotic classes without the data from Portugal. Significant trends are highlighted with a red R^2 and p-value (p<0.05). Shaded cells indicate summary classes of antibiotics – those that are sums of other columns (“All beta-lactams” (J01C_D), “beta-lactams” (J01C), “other beta-lactams” (J01D), see Table S5). Shaded areas around the blue trend line are the 95% confidence level interval for predictions from the linear model.

**Other antibiotics and MRSA infection incidence**

We fitted (a) linear models (y~x) and (b) exponential models (log(y) ~ x) to total antibiotic usage (x) and MRSA infection (y) across all antibiotic groupings available for the up to 29 countries in the ESAC-Net data.

*Quinolones*

There is a significant positive correlation between the quinolone (J01M) class of antibiotic use across the community and hospital with MRSA infection incidence (Figure S20, linear and Figure S21, exponential). This is driven by the use of fluoroquinolones (J01MA), specifically those in the second generation of quinolones (J01M_scnd) such as ciprofloxacin, enoxacin, lomefloxacin, norfloxacin and ofloxacin. Removing the data from Portugal did not affect these relationships under a linear model (Figure S22) but assuming an exponential model there is also a significant correlation between 1^st^ generation quinolone use and MRSA infection incidence (Figure S23).

Figure S20: Correlation between quinolone antibiotic usage (x axis) and MRSA infection incidence (y axis) assuming a linear model. Significant trends are highlighted with a red R^2 and p-value (p<0.05). Shaded cells indicate summary classes of antibiotics – those that are sums of other columns (“All quinolones” (J01M), “fluoroquinolones” (J01MA), see Table S5). Shaded areas around the blue trend line are the 95% confidence level interval for predictions from the linear model.

Figure S21: Correlation between quinolone antibiotic usage (x axis) and MRSA infection incidence (y axis) assuming an exponential model. Significant trends are highlighted with a red R^2 and p-value (p<0.05). Shaded cells indicate summary classes of antibiotics – those that are sums of other columns (“All quinolones” (J01M), “fluoroquinolones” (J01MA), see Table S5). Shaded areas around the blue trend line are the 95% confidence level interval for predictions from the linear model.

Figure S22: Correlation between quinolone antibiotic usage (x axis) and MRSA infection incidence (y axis) without the data from Portugal assuming a linear model. Significant trends are highlighted with a red R^2 and p-value (p<0.05). Shaded cells indicate summary classes of antibiotics – those that are sums of other columns (“All quinolones” (J01M), “fluoroquinolones” (J01MA), see Table S5). Shaded areas around the blue trend line are the 95% confidence level interval for predictions from the linear model.

l

Figure S23: Correlation between quinolone antibiotic usage (x axis) and MRSA infection incidence (y axis) without the data from Portugal assuming an exponential model. Significant trends are highlighted with a red R^2 and p-value (p<0.05). Shaded cells indicate summary classes of antibiotics – those that are sums of other columns (“All quinolones” (J01M), “fluoroquinolones” (J01MA), see Table S5). Shaded areas around the blue trend line are the 95% confidence level interval for predictions from the linear model.

*Macrolides*

There is a significant positive correlation between the macrolides, lincosamides and streptogramins (J01F) class of antibiotic use across the community and hospital with MRSA infection incidence (Figure S24 & S25, linear with and without out Portugal, and exponential Figure S26 & S27). This is driven by the use of macrolides (J01FA), particularly those with intermediate- (J01FA_intr) or long- (J01FA_long) acting action under a linear or exponential model respectively.

Figure S24: Correlation between macrolide antibiotic usage (x axis) and MRSA infection incidence (y axis) assuming a linear model. From left to right are “macrolides, lincosamides and streptogramins” (“J01F”) and “macrolides” (“J01FA") - the summary class totals. Following this are the constituent antibiotic groups, in different sub groupings. Significant trends are highlighted with a red R^2 and p-value (p<0.05). Shaded cells indicate summary classes of antibiotics – those that are sums of other columns (see Table S5). Shaded areas around the blue trend line are the 95% confidence level interval for predictions from the linear model.

Figure S25: Correlation between macrolide antibiotic usage (x axis) and MRSA infection incidence (y axis) assuming a linear model without the data from Portugal. From left to right are “macrolides, lincosamides and streptogramins” (“J01F”) and “macrolides” (“J01FA") - the summary class totals. Following this are the constituent antibiotic groups, in different sub groupings. Significant trends are highlighted with a red R^2 and p-value (p<0.05). Shaded cells indicate summary classes of antibiotics – those that are sums of other columns (see Table S5). Shaded areas around the blue trend line are the 95% confidence level interval for predictions from the linear model.

Figure S26: Correlation between macrolide antibiotic usage (x axis) and MRSA infection incidence (y axis) assuming an exponential model. From left to right are “macrolides, lincosamides and streptogramins” (“J01F”) and “macrolides” (“J01FA") - the summary class totals. Following this are the constituent antibiotic groups, in different sub groupings. Significant trends are highlighted with a red R^2 and p-value (p<0.05). Shaded cells indicate summary classes of antibiotics – those that are sums of other columns (see Table S5). Shaded areas around the blue trend line are the 95% confidence level interval for predictions from the linear model.

Figure S27: Correlation between macrolide antibiotic usage (x axis) and MRSA infection incidence (y axis) assuming an exponential mode without the data from Portugal. From left to right are “macrolides, lincosamides and streptogramins” (“J01F”) and “macrolides” (“J01FA"). Following this are the constituent antibiotic groups, in different sub groupings. Significant trends are highlighted with a red R^2 and p-value (p<0.05). Shaded cells indicate summary classes of antibiotics – those that are sums of other columns (see Table S5). Shaded areas around the blue trend line are the 95% confidence level interval for predictions from the linear model.

*Tetracycline*

There was not a significant trend for the association with tetracycline antibiotics (Figure S28), but a suggestion of a negative correlation was seen.

**

Figure S28: Correlation between tetracycline antibiotic usage (x axis) and MRSA infection incidence (y axis) assuming a linear model. From left to right are “macrolides, lincosamides and streptogramins” (“J01F”) and “macrolides” (“J01FA") - the summary class totals. Following this are the constituent antibiotic groups, in different sub groupings. Significant trends are highlighted with a red R^2 and p-value (p<0.05). Shaded cells indicate summary classes of antibiotics – those that are sums of other columns (see Table S5). Shaded areas around the blue trend line are the 95% confidence level interval for predictions from the linear model.

*Trimethoprim*

A significant link between Intermediate-active sulphonamides and MRSA infection incidence (Figure S29) was driven by the data from Portugal – excluding this outlier no significant trends in the usage of sulphonamides and trimethoprim was left (Figure S30).

**

Figure S29: Correlation between trimethoprim antibiotic usage (x axis) and MRSA infection incidence (y axis) assuming a linear model. From left to right are “sulphonamides and trimethoprim” (“J01E”) and at the end “Combinations of sulphonamides and trimethoprim, incl. derivatives” (“J01EE") - the summary class totals. In the middle are the constituent antibiotic groups, in different sub groupings. Significant trends are highlighted with a red R^2 and p-value (p<0.05). Shaded cells indicate summary classes of antibiotics – those that are sums of other columns (see Table S5). Shaded areas around the blue trend line are the 95% confidence level interval for predictions from the linear model.

**

Figure S30: Correlation between trimethoprim antibiotic usage (x axis) and MRSA infection incidence (y axis) without data from Portugal assuming a linear model. From left to right are “sulphonamides and trimethoprim” (“J01E”) and at the end “Combinations of sulphonamides and trimethoprim, incl. derivatives” (“J01EE") - the summary class totals. In the middle are the constituent antibiotic groups, in different sub groupings. Significant trends are highlighted with a red R^2 and p-value (p<0.05). Shaded cells indicate summary classes of antibiotics – those that are sums of other columns (see Table S5). Shaded areas around the blue trend line are the 95% confidence level interval for predictions from the linear model.

*Aminoglycoside*

A significant correlation between aminoglycoside use in the hospital and MRSA infection incidence under a linear model (Figure S31) is driven by high usage in Italy. Without the Portugal data point (where few aminoglycosides are used in the community) there is also a significant trend for combined community and hospital use and MRSA infection incidence. However, this is driven by high aminoglycoside use in Romania (community) and Italy (hospital): the significant trend is lost when data from these countries and Portugal are removed (Figure S32).

**

Figure S31: Correlation between aminoglycoside antibiotic usage (x axis) and MRSA infection incidence (y axis) assuming a linear model with country labels. Significant trends are highlighted with a red R^2 and p-value (p<0.05). Shaded cells indicate summary classes of antibiotics – those that are sums of other columns (see Table S5). Shaded areas around the blue trend line are the 95% confidence level interval for predictions from the linear model.

**

Figure S32: Correlation between aminoglycoside antibiotic usage (x axis) and MRSA infection incidence (y axis) assuming a linear model without the data from Portugal, Romania and Italy. Significant trends are highlighted with a red R^2 and p-value (p<0.05). Shaded cells indicate summary classes of antibiotics – those that are sums of other columns (see Table S5). Shaded areas around the blue trend line are the 95% confidence level interval for predictions from the linear model.

**Supplementary Table S6**

A linear (y ~ x) and exponential (log(y) ~ x) model. * indicates a *p*-value for the value (the coefficient of the trend) less than 0.05, ** a *p*-value less than 0.01.

| **Setting** | **Drug** | **Model** | **Intercept** | **Value** | **R^2^** | **AIC** | ***p*-value** | **significant** |
| --- | --- | --- | --- | --- | --- | --- | --- | --- |
| Community | 1G_cephalosporins | linear | 24.5764 | -6.3564 | 0.0103 | 270.958 | 0.6 |  |
| Hospital | 1G_cephalosporins | linear | 26.4672 | -57.0897 | 0.016 | 219.646 | 0.565 |  |
| Community & Hospital | 1G_cephalosporins | linear | 25.0717 | -6.8316 | 0.0136 | 270.863 | 0.547 |  |
| Community | 1G_cephalosporins | exponential | 2.6954 | -0.3341 | 0.0127 | 94.0153 | 0.561 |  |
| Hospital | 1G_cephalosporins | exponential | 2.6614 | -2.5082 | 0.0141 | 78.7895 | 0.589 |  |
| Community & Hospital | 1G_cephalosporins | exponential | 2.7304 | -0.3916 | 0.0199 | 93.8043 | 0.466 |  |
| Community | 1G_quinolones | linear | 20.4862 | 19.4484 | 0.0355 | 235.658 | 0.367 |  |
| Hospital | 1G_quinolones | linear | 28.4137 | -486.1238 | 0.0188 | 184.668 | 0.576 |  |
| Community & Hospital | 1G_quinolones | linear | 20.5778 | 18.6562 | 0.0334 | 235.713 | 0.382 |  |
| Community | 1G_quinolones | exponential | 2.351 | 1.6076 | 0.1239 | 77.3727 | 0.0844 |  |
| Hospital | 1G_quinolones | exponential | 2.5564 | 7.4807 | 0.0023 | 66.151 | 0.845 |  |
| Community & Hospital | 1G_quinolones | exponential | 2.3522 | 1.5677 | 0.1203 | 77.4759 | 0.0894 |  |
| Community | 2G_cephalosporins | linear | 19.396 | 2.2863 | 0.0269 | 270.468 | 0.395 |  |
| Hospital | 2G_cephalosporins | linear | 29.9208 | -49.2948 | 0.0841 | 217.997 | 0.179 |  |
| Community & Hospital | 2G_cephalosporins | linear | 19.8428 | 1.8795 | 0.0188 | 270.708 | 0.478 |  |
| Community | 2G_cephalosporins | exponential | 2.1845 | 0.264 | 0.1598 | 89.3359 | 0.0317 | * |
| Hospital | 2G_cephalosporins | exponential | 2.7128 | -1.513 | 0.0363 | 78.2674 | 0.384 |  |
| Community & Hospital | 2G_cephalosporins | exponential | 2.195 | 0.2401 | 0.1369 | 90.1173 | 0.0482 | * |
| Community | 2G_quinolones | linear | -0.2738 | 16.83 | 0.3511 | 250.698 | 0.000893 | ** |
| Hospital | 2G_quinolones | linear | -2.3478 | 131.3422 | 0.2331 | 205.637 | 0.0228 | * |
| Community & Hospital | 2G_quinolones | linear | -2.6497 | 16.724 | 0.3727 | 249.751 | 0.000561 | ** |
| Community | 2G_quinolones | exponential | 1.3537 | 0.8995 | 0.443 | 75.8815 | 0.000111 | ** |
| Hospital | 2G_quinolones | exponential | 1.2045 | 6.6703 | 0.2726 | 69.9034 | 0.0127 | * |
| Community & Hospital | 2G_quinolones | exponential | 1.2645 | 0.8698 | 0.4453 | 75.7653 | 0.000105 | ** |
| Community | 3G_cephalosporins | linear | 13.8149 | 27.349 | 0.3512 | 258.711 | 0.000705 | ** |
| Hospital | 3G_cephalosporins | linear | 21.9623 | 2.5468 | 1e-04 | 220.014 | 0.956 |  |
| Community & Hospital | 3G_cephalosporins | linear | 12.3791 | 23.4119 | 0.2972 | 261.033 | 0.00223 | ** |
| Community | 3G_cephalosporins | exponential | 2.1842 | 1.2785 | 0.3419 | 82.2508 | 0.000864 | ** |
| Hospital | 3G_cephalosporins | exponential | 2.3766 | 0.6912 | 0.005 | 79.0027 | 0.75 |  |
| Community & Hospital | 3G_cephalosporins | exponential | 2.1255 | 1.0762 | 0.2797 | 84.8712 | 0.00318 | ** |
| Community | 3G_quinolones | linear | 20.6221 | 19.6945 | 0.0309 | 261.93 | 0.371 |  |
| Hospital | 3G_quinolones | linear | 29.8545 | -437.53 | 0.0791 | 209.663 | 0.205 |  |
| Community & Hospital | 3G_quinolones | linear | 20.8326 | 16.8422 | 0.0243 | 262.118 | 0.428 |  |
| Community | 3G_quinolones | exponential | 2.3809 | 1.638 | 0.0943 | 89.4937 | 0.112 |  |
| Hospital | 3G_quinolones | exponential | 2.5558 | -4.4102 | 0.0036 | 76.826 | 0.79 |  |
| Community & Hospital | 3G_quinolones | exponential | 2.3841 | 1.4867 | 0.0837 | 89.8183 | 0.135 |  |
| Community | 4G_cephalosporin | linear | 20.7955 | 6088.74 | 0.0717 | 269.103 | 0.16 |  |
| Hospital | 4G_cephalosporin | linear | 22.8253 | -134.5673 | 6e-04 | 220.003 | 0.91 |  |
| Community & Hospital | 4G_cephalosporin | linear | 22.9253 | 81.6074 | 3e-04 | 271.251 | 0.931 |  |
| Community | 4G_cephalosporin | exponential | 2.5031 | 303.5589 | 0.0793 | 91.9889 | 0.139 |  |
| Hospital | 4G_cephalosporin | exponential | 2.3121 | 47.0492 | 0.0347 | 78.3049 | 0.395 |  |
| Community & Hospital | 4G_cephalosporin | exponential | 2.5184 | 32.1941 | 0.0195 | 93.8146 | 0.47 |  |
| Community | aminoglycosides | linear | 22.3542 | 22.0067 | 0.0042 | 271.137 | 0.738 |  |
| Hospital | aminoglycosides | linear | 5.2042 | 256.6558 | 0.2434 | 213.603 | 0.0168 | * |
| Community & Hospital | aminoglycosides | linear | 15.8647 | 80.6701 | 0.0796 | 268.854 | 0.138 |  |
| Community | aminoglycosides | exponential | 2.5223 | 2.6408 | 0.027 | 93.5915 | 0.394 |  |
| Hospital | aminoglycosides | exponential | 1.7149 | 11.4606 | 0.2221 | 73.341 | 0.0232 | * |
| Community & Hospital | aminoglycosides | exponential | 2.2633 | 3.9553 | 0.0852 | 91.8026 | 0.124 |  |
| Community | amphenicols | linear | 20.7725 | 527.2924 | 0.0851 | 268.681 | 0.125 |  |
| Hospital | amphenicols | linear | 21.1605 | 7339.5001 | 0.0048 | 219.906 | 0.753 |  |
| Community & Hospital | amphenicols | linear | 21.1009 | 509.6842 | 0.0802 | 244.319 | 0.161 |  |
| Community | amphenicols | exponential | 2.5236 | 21.5626 | 0.0634 | 92.4876 | 0.188 |  |
| Hospital | amphenicols | exponential | 2.3242 | 967.5341 | 0.0383 | 78.2192 | 0.371 |  |
| Community & Hospital | amphenicols | exponential | 2.4738 | 22.4534 | 0.0695 | 86.0062 | 0.193 |  |
| Community | antibacterials_for_systemic | linear | -8.9844 | 1.7826 | 0.1699 | 265.859 | 0.0263 | * |
| Hospital | antibacterials_for_systemic | linear | 8.0037 | 8.2151 | 0.0177 | 219.608 | 0.546 |  |
| Community & Hospital | antibacterials_for_systemic | linear | -10.671 | 1.7423 | 0.1657 | 266.005 | 0.0284 | * |
| Community | antibacterials_for_systemic | exponential | 0.374 | 0.1246 | 0.3697 | 81.0017 | 0.000467 | ** |
| Hospital | antibacterials_for_systemic | exponential | 1.4161 | 0.6096 | 0.0445 | 78.0703 | 0.334 |  |
| Community & Hospital | antibacterials_for_systemic | exponential | 0.3307 | 0.1179 | 0.3382 | 82.4167 | 0.000938 | ** |
| Community | b-lactam | linear | -3.3503 | 3.5998 | 0.1756 | 265.659 | 0.0236 | * |
| Hospital | b-lactam | linear | 17.4409 | 8.0904 | 0.0055 | 219.891 | 0.737 |  |
| Community & Hospital | b-lactam | linear | -3.4871 | 3.4005 | 0.1651 | 266.028 | 0.0287 | * |
| Community | b-lactam | exponential | 1.0332 | 0.2156 | 0.2805 | 84.8384 | 0.00313 | ** |
| Hospital | b-lactam | exponential | 2.2432 | 0.3912 | 0.0059 | 78.9817 | 0.728 |  |
| Community & Hospital | b-lactam | exponential | 1.076 | 0.1971 | 0.2471 | 86.1546 | 0.00608 | ** |
| Community | blactamase_inhibitors | linear | 23.4154 | -218845 | 0.0026 | 271.183 | 0.792 |  |
| Hospital | blactamase_inhibitors | linear | 18.7255 | 39635 | 0.4476 | 206.367 | 0.000482 | ** |
| Community & Hospital | blactamase_inhibitors | linear | 20.3365 | 38836 | 0.4027 | 256.315 | 0.000218 | ** |
| Community | blactamase_inhibitors | exponential | 2.6152 | 6970 | 0.0012 | 94.3517 | 0.86 |  |
| Hospital | blactamase_inhibitors | exponential | 2.3828 | 1067 | 0.1486 | 75.4169 | 0.0693 |  |
| Community & Hospital | blactamase_inhibitors | exponential | 2.5498 | 989 | 0.1165 | 90.7944 | 0.07 |  |
| Community & Hospital | blactamase_resistant_pencillins | linear | 27.6148 | -7.0592 | 0.0817 | 531.574 | 0.0296 | * |
| Community | blactamase_resistant_pencillins | linear | 25.0276 | -5.3054 | 0.017 | 270.762 | 0.5 |  |
| Hospital | blactamase_resistant_pencillins | linear | 28.3878 | -66.7308 | 0.0692 | 218.368 | 0.225 |  |
| Community & Hospital | blactamase_resistant_pencillins | exponential | 2.9155 | -0.4675 | 0.1596 | 172.685 | 0.00189 | ** |
| Community | blactamase_resistant_pencillins | exponential | 2.7715 | -0.4302 | 0.0498 | 92.9042 | 0.245 |  |
| Hospital | blactamase_resistant_pencillins | exponential | 2.8902 | -4.5257 | 0.1457 | 75.4959 | 0.0723 |  |
| Community | blactamase_sensitive_penicillins | linear | 29.8214 | -8.3772 | 0.1487 | 266.59 | 0.0388 | * |
| Hospital | blactamase_sensitive_penicillins | linear | 29.7027 | -130.4878 | 0.1413 | 216.512 | 0.0771 |  |
| Community | blactamase_sensitive_penicillins | exponential | 3.0358 | -0.5221 | 0.2573 | 85.7585 | 0.00497 | ** |
| Hospital | blactamase_sensitive_penicillins | exponential | 2.9975 | -9.1713 | 0.3196 | 70.2616 | 0.00494 | ** |
| Community | carbapenems | linear | 21.4904 | 187.2892 | 0.0344 | 270.245 | 0.336 |  |
| Hospital | carbapenems | linear | -3.6525 | 556.2122 | 0.2395 | 213.719 | 0.0178 | * |
| Community & Hospital | carbapenems | linear | 8.7533 | 303.141 | 0.1575 | 258.288 | 0.0365 | * |
| Community | carbapenems | exponential | 2.5056 | 12.8817 | 0.0725 | 92.2048 | 0.158 |  |
| Hospital | carbapenems | exponential | 1.2818 | 25.6411 | 0.233 | 73.0169 | 0.0197 | * |
| Community & Hospital | carbapenems | exponential | 1.9727 | 13.2566 | 0.1349 | 88.1128 | 0.0545 |  |
| Community | Combination penicillins (incl. blactamase inhibitors) | linear | -3.3717 | 7.7343 | 0.5467 | 248.317 | 4.61e-06 |  |
| Hospital |  | linear | -4.8744 | 78.9248 | 0.2975 | 211.896 | 0.00711 | ** |
| Community & Hospital |  | linear | -4.6492 | 7.507 | 0.5535 | 247.878 | 3.74e-06 |  |
| Community |  | exponential | 1.1865 | 0.4181 | 0.7117 | 58.3158 | 9.07e-09 |  |
| Hospital |  | exponential | 0.7279 | 5.0814 | 0.5644 | 60.0053 | 3.61e-05 |  |
| Community & Hospital |  | exponential | 1.1215 | 0.4048 | 0.7167 | 57.8103 | 7.15e-09 |  |
| Community | fluoroquinolones | linear | 0.7049 | 12.9014 | 0.3308 | 259.61 | 0.0011 | ** |
| Hospital |  | linear | -1.1292 | 112.1036 | 0.185 | 215.311 | 0.0405 | * |
| Community & Hospital |  | linear | -1.6296 | 13.0034 | 0.3492 | 258.802 | 0.000737 | ** |
| Community |  | exponential | 1.3591 | 0.7249 | 0.4653 | 76.2319 | 4.6e-05 |  |
| Hospital |  | exponential | 1.1966 | 6.1307 | 0.2533 | 72.3994 | 0.0144 | * |
| Community & Hospital |  | exponential | 1.2556 | 0.7162 | 0.4718 | 75.8739 | 3.87e-05 |  |
| Community | glycopeptides | linear | 21.2007 | 365.4059 | 0.0379 | 270.139 | 0.312 |  |
| Hospital | glycopeptides | linear | 2.3261 | 550.6752 | 0.2281 | 214.062 | 0.0212 | * |
| Community & Hospital | glycopeptides | linear | 11.3525 | 345.7477 | 0.1537 | 266.419 | 0.0354 | * |
| Community | glycopeptides | exponential | 2.4889 | 24.539 | 0.0761 | 92.0894 | 0.147 |  |
| Hospital | glycopeptides | exponential | 1.3867 | 30.083 | 0.3115 | 70.5307 | 0.00564 | ** |
| Community & Hospital | glycopeptides | exponential | 2.0731 | 16.0478 | 0.1475 | 89.7573 | 0.0397 | * |
| Community | imadazole | linear | 23.0841 | 5.1742 | 1e-04 | 271.255 | 0.952 |  |
| Hospital | imadazole | linear | 34.1617 | -194.4192 | 0.0704 | 218.339 | 0.221 |  |
| Community & Hospital | imadazole | linear | 26.9909 | -52.617 | 0.0206 | 262.504 | 0.466 |  |
| Community | imadazole | exponential | 2.6385 | -0.7898 | 0.0014 | 94.3452 | 0.847 |  |
| Hospital | imadazole | exponential | 2.6392 | -2.6142 | 0.0058 | 78.9825 | 0.729 |  |
| Community & Hospital | imadazole | exponential | 2.7881 | -2.5531 | 0.0218 | 91.5541 | 0.454 |  |
| Community | intermediate_acting_macrolides | linear | 14.5524 | 5.5021 | 0.0754 | 260.611 | 0.157 |  |
| Hospital | intermediate_acting_macrolides | linear | 14.4439 | 124.4167 | 0.0688 | 209.907 | 0.238 |  |
| Community & Hospital | intermediate_acting_macrolides | linear | 14.4014 | 5.4189 | 0.0765 | 260.578 | 0.154 |  |
| Community | intermediate_acting_macrolides | exponential | 1.9172 | 0.4327 | 0.2061 | 85.8047 | 0.0152 | * |
| Hospital | intermediate_acting_macrolides | exponential | 1.9544 | 7.8371 | 0.1238 | 73.9978 | 0.108 |  |
| Community & Hospital | intermediate_acting_macrolides | exponential | 1.9159 | 0.42 | 0.2031 | 85.9107 | 0.0161 | * |
| Community | intermediate_acting_sulfonamides | linear | 21.4431 | 793.5728 | 0.0292 | 270.4 | 0.376 |  |
| Hospital | intermediate_acting_sulfonamides | linear | 16.0065 | 4390.6944 | 0.3164 | 211.269 | 0.00521 | ** |
| Community & Hospital | intermediate_acting_sulfonamides | linear | 16.4118 | 1862.0783 | 0.1952 | 240.601 | 0.0238 | * |
| Community | intermediate_acting_sulfonamides | exponential | 2.5047 | 53.5029 | 0.0591 | 92.6197 | 0.204 |  |
| Hospital | intermediate_acting_sulfonamides | exponential | 2.2655 | 148.8064 | 0.1663 | 74.9331 | 0.0534 |  |
| Community & Hospital | intermediate_acting_sulfonamides | exponential | 2.2565 | 86.248 | 0.1892 | 81.8613 | 0.0264 | * |
| Community | lincosamides | linear | 31.5079 | -29.5793 | 0.0812 | 268.804 | 0.134 |  |
| Hospital | lincosamides | linear | 17.345 | 137.2126 | 0.0173 | 219.616 | 0.55 |  |
| Community & Hospital | lincosamides | linear | 31.9335 | -28.205 | 0.0753 | 268.99 | 0.15 |  |
| Community | lincosamides | exponential | 2.6778 | -0.1968 | 0.0016 | 94.3395 | 0.837 |  |
| Hospital | lincosamides | exponential | 2.1492 | 9.0867 | 0.0347 | 78.3048 | 0.395 |  |
| Community & Hospital | lincosamides | exponential | 2.6808 | -0.1882 | 0.0015 | 94.3426 | 0.842 |  |
| Community | long_acting_macrolides | linear | 5.7342 | 17.5947 | 0.1637 | 257.803 | 0.0327 | * |
| Hospital | long_acting_macrolides | linear | 7.6025 | 542.5709 | 0.2615 | 204.809 | 0.015 | * |
| Community & Hospital | long_acting_macrolides | linear | 5.2174 | 17.7048 | 0.1722 | 257.517 | 0.0281 | * |
| Community | long_acting_macrolides | exponential | 1.4197 | 1.1911 | 0.3314 | 80.9972 | 0.00135 | ** |
| Hospital | long_acting_macrolides | exponential | 1.8591 | 22.2525 | 0.1994 | 72.0134 | 0.0372 | * |
| Community & Hospital | long_acting_macrolides | exponential | 1.415 | 1.1694 | 0.3318 | 80.9774 | 0.00134 | ** |
| Community | macrolides | linear | 6.3199 | 6.0172 | 0.1335 | 267.102 | 0.0513 |  |
| Hospital | macrolides | linear | 6.8903 | 138.7363 | 0.127 | 216.892 | 0.095 |  |
| Community & Hospital | macrolides | linear | 5.9436 | 5.9651 | 0.1366 | 266.999 | 0.0484 | * |
| Community | macrolides | exponential | 1.366 | 0.4482 | 0.33 | 82.7727 | 0.00112 | ** |
| Hospital | macrolides | exponential | 1.58 | 8.0825 | 0.1973 | 74.0609 | 0.0337 | * |
| Community & Hospital | macrolides | exponential | 1.3585 | 0.4372 | 0.3269 | 82.9054 | 0.00119 | ** |
| Community | Macrolides, lincosamides, streptogramins | linear | 6.4453 | 5.3713 | 0.1123 | 267.806 | 0.0756 |  |
| Hospital |  | linear | 2.1966 | 134.8265 | 0.1498 | 216.285 | 0.0681 |  |
| Community & Hospital |  | linear | 5.8133 | 5.3714 | 0.1169 | 267.653 | 0.0694 |  |
| Community |  | exponential | 1.2553 | 0.4386 | 0.3334 | 82.6267 | 0.00104 | ** |
| Hospital |  | exponential | 1.2967 | 7.9207 | 0.2366 | 72.9086 | 0.0186 | * |
| Community & Hospital |  | exponential | 1.2349 | 0.4289 | 0.3321 | 82.6792 | 0.00107 | ** |
| Community | monobactams | linear | 25.1981 | -4315 | 0.0302 | 270.371 | 0.368 |  |
| Hospital | monobactams | linear | 20.0678 | 2037 | 0.0207 | 219.536 | 0.512 |  |
| Community & Hospital | monobactams | linear | 20.992 | 575 | 0.0023 | 244.793 | 0.815 |  |
| Community | monobactams | exponential | 2.7634 | -302 | 0.0662 | 92.4005 | 0.178 |  |
| Hospital | monobactams | exponential | 2.2702 | 188 | 0.0807 | 77.1814 | 0.189 |  |
| Community & Hospital | monobactams | exponential | 2.446 | 54 | 0.0093 | 85.6092 | 0.639 |  |
| Community | nitrofuran | linear | 24.1002 | -1.3024 | 0.002 | 271.201 | 0.817 |  |
| Hospital | nitrofuran | linear | 24.4753 | -85.836 | 0.009 | 219.81 | 0.667 |  |
| Community & Hospital | nitrofuran | linear | 24.1698 | -1.3623 | 0.0023 | 271.192 | 0.804 |  |
| Community | nitrofuran | exponential | 2.6312 | -0.0124 | 1e-04 | 94.3836 | 0.963 |  |
| Hospital | nitrofuran | exponential | 2.477 | 0.134 | 0 | 79.1166 | 0.989 |  |
| Community & Hospital | nitrofuran | exponential | 2.6343 | -0.0165 | 2e-04 | 94.3816 | 0.95 |  |
| Community | other_aminoglycosides | linear | 22.3818 | 22.125 | 0.0035 | 271.157 | 0.76 |  |
| Hospital | other_aminoglycosides | linear | 5.3003 | 256.8886 | 0.2437 | 213.594 | 0.0167 | * |
| Community & Hospital | other_aminoglycosides | linear | 15.4587 | 86.8572 | 0.0827 | 268.755 | 0.13 |  |
| Community | other_aminoglycosides | exponential | 2.5181 | 2.8597 | 0.0261 | 93.6194 | 0.403 |  |
| Hospital | other_aminoglycosides | exponential | 1.7254 | 11.3772 | 0.2187 | 73.4394 | 0.0244 | * |
| Community & Hospital | other_aminoglycosides | exponential | 2.2518 | 4.1649 | 0.0847 | 91.8186 | 0.126 |  |
| Community | other_blactams | linear | 14.6398 | 3.8313 | 0.0888 | 268.563 | 0.116 |  |
| Hospital | other_blactams | linear | 31.6961 | -21.8766 | 0.0325 | 219.258 | 0.411 |  |
| Community & Hospital | other_blactams | linear | 14.5059 | 3.3786 | 0.0718 | 269.097 | 0.16 |  |
| Community | other_blactams | exponential | 1.9268 | 0.3118 | 0.2619 | 85.5812 | 0.00455 | ** |
| Hospital | other_blactams | exponential | 2.6461 | -0.3879 | 0.0047 | 79.0091 | 0.757 |  |
| Community & Hospital | other_blactams | exponential | 1.8981 | 0.2819 | 0.2227 | 87.0804 | 0.00975 | ** |
| Community | Other cephalosporins and penems | linear | 22.9315 | 10516 | 0.0015 | 271.214 | 0.84 |  |
| Hospital |  | linear | 22.9866 | -10048 | 0.0052 | 219.898 | 0.745 |  |
| Community & Hospital |  | linear | 22.5995 | -4898 | 0.0015 | 253.373 | 0.85 |  |
| Community |  | exponential | 2.5772 | 1846 | 0.0212 | 93.7654 | 0.451 |  |
| Hospital |  | exponential | 2.4938 | -212 | 0.0011 | 79.0926 | 0.883 |  |
| Community & Hospital |  | exponential | 2.5414 | 197 | 0.0011 | 88.592 | 0.872 |  |
| Community | other_quinolones | linear | 24.6454 | -344 | 0.0191 | 270.701 | 0.475 |  |
| Hospital | other_quinolones | linear | 23.9007 | -13257 | 0.0336 | 219.23 | 0.402 |  |
| Community & Hospital | other_quinolones | linear | 24.7287 | -447 | 0.0323 | 270.306 | 0.351 |  |
| Community | other_quinolones | exponential | 2.6706 | -11 | 0.0093 | 94.1154 | 0.619 |  |
| Hospital | other_quinolones | exponential | 2.5364 | -477 | 0.02 | 78.6528 | 0.52 |  |
| Community & Hospital | other_quinolones | exponential | 2.6887 | -19 | 0.0266 | 93.6028 | 0.398 |  |
| Community | penicillins_with_extended_spectrum | linear | 23.8042 | -0.2205 | 2e-04 | 271.253 | 0.939 |  |
| Hospital | penicillins_with_extended_spectrum | linear | 29.8229 | -65.2031 | 0.0639 | 218.498 | 0.244 |  |
| Community & Hospital | penicillins_with_extended_spectrum | linear | 24.4461 | -0.4363 | 9e-04 | 271.233 | 0.877 |  |
| Community | penicillins_with_extended_spectrum | exponential | 2.3353 | 0.1029 | 0.0215 | 93.755 | 0.448 |  |
| Hospital | penicillins_with_extended_spectrum | exponential | 2.9763 | -4.3244 | 0.1287 | 75.9482 | 0.0928 |  |
| Community & Hospital | penicillins_with_extended_spectrum | exponential | 2.3887 | 0.0811 | 0.0139 | 93.979 | 0.542 |  |
| Community | polymyxins | linear | 22.3673 | 111.0913 | 0.0017 | 271.211 | 0.834 |  |
| Hospital | polymyxins | linear | 13.7861 | 1472.2414 | 0.2335 | 213.902 | 0.0195 | * |
| Community & Hospital | polymyxins | linear | 16.2489 | 563.8457 | 0.0866 | 260.552 | 0.129 |  |
| Community | polymyxins | exponential | 2.509 | 15.3429 | 0.014 | 93.976 | 0.54 |  |
| Hospital | polymyxins | exponential | 2.1018 | 65.1184 | 0.209 | 73.7231 | 0.0283 | * |
| Community & Hospital | polymyxins | exponential | 2.2548 | 28.3262 | 0.0978 | 89.2874 | 0.105 |  |
| Community | Quinolones | linear | 0.7002 | 12.8736 | 0.3293 | 259.678 | 0.00114 | ** |
| Hospital | Quinolones | linear | -1.0879 | 111.8367 | 0.1844 | 215.33 | 0.0409 | * |
| Community & Hospital | Quinolones | linear | -1.6471 | 12.9821 | 0.348 | 258.857 | 0.000758 | ** |
| Community | Quinolones | exponential | 1.357 | 0.7244 | 0.4644 | 76.2807 | 4.7e-05 |  |
| Hospital | Quinolones | exponential | 1.1979 | 6.1205 | 0.2527 | 72.4163 | 0.0145 | * |
| Community & Hospital | Quinolones | exponential | 1.2528 | 0.7159 | 0.4714 | 75.8997 | 3.91e-05 |  |
| Community | short_acting_macrolides | linear | 25.2293 | -12.5716 | 0.0045 | 262.682 | 0.735 |  |
| Hospital | short_acting_macrolides | linear | 21.279 | 308.0989 | 0.0059 | 202.372 | 0.741 |  |
| Community & Hospital | short_acting_macrolides | linear | 25.1594 | -11.4514 | 0.0039 | 262.697 | 0.752 |  |
| Community | short_acting_macrolides | exponential | 2.7752 | -1.1333 | 0.016 | 91.8145 | 0.521 |  |
| Hospital | short_acting_macrolides | exponential | 2.5939 | -4.5876 | 6e-04 | 73.1068 | 0.915 |  |
| Community & Hospital | short_acting_macrolides | exponential | 2.7798 | -1.1145 | 0.0164 | 91.8046 | 0.516 |  |
| Community | short_acting_sulphonamides | linear | 23.9109 | -113.7814 | 0.0242 | 270.547 | 0.42 |  |
| Hospital | short_acting_sulphonamides | linear | 23.8327 | -6223.925 | 0.0368 | 219.155 | 0.381 |  |
| Community & Hospital | short_acting_sulphonamides | linear | 23.7892 | -112.196 | 0.0257 | 227.954 | 0.454 |  |
| Community | short_acting_sulphonamides | exponential | 2.6755 | -8.3659 | 0.0584 | 92.641 | 0.207 |  |
| Hospital | short_acting_sulphonamides | exponential | 2.5548 | -311.4905 | 0.0422 | 78.1262 | 0.347 |  |
| Community & Hospital | short_acting_sulphonamides | exponential | 2.587 | -7.7292 | 0.0546 | 80.7685 | 0.272 |  |
| Community | steroid_antibacterials | linear | 19.918 | 529.0515 | 0.0935 | 268.411 | 0.107 |  |
| Hospital | steroid_antibacterials | linear | 19.3439 | 3450.5306 | 0.0471 | 218.909 | 0.32 |  |
| Community & Hospital | steroid_antibacterials | linear | 18.981 | 550.8718 | 0.1053 | 243.366 | 0.106 |  |
| Community | steroid_antibacterials | exponential | 2.496 | 20.4546 | 0.0623 | 92.5208 | 0.192 |  |
| Hospital | steroid_antibacterials | exponential | 2.3712 | 125.4087 | 0.0284 | 78.4531 | 0.442 |  |
| Community & Hospital | steroid_antibacterials | exponential | 2.3967 | 22.7176 | 0.081 | 85.106 | 0.159 |  |
| Community | streptogramins | linear | 22.2982 | 27.5099 | 0.0404 | 270.062 | 0.296 |  |
| Hospital | streptogramins | linear | 21.1657 | 759.3244 | 0.0466 | 218.918 | 0.322 |  |
| Community & Hospital | streptogramins | linear | 21.172 | 27.6461 | 0.0469 | 218.911 | 0.321 |  |
| Community | streptogramins | exponential | 2.5778 | 1.3803 | 0.0453 | 93.0403 | 0.267 |  |
| Hospital | streptogramins | exponential | 2.4164 | 41.1737 | 0.0628 | 77.6258 | 0.249 |  |
| Community & Hospital | streptogramins | exponential | 2.4169 | 1.4943 | 0.0628 | 77.6259 | 0.249 |  |
| Community | sulfonamides_and_trimethoprim | linear | 24.9509 | -4.3774 | 0.0018 | 271.206 | 0.826 |  |
| Hospital | sulfonamides_and_trimethoprim | linear | 23.9953 | -28.5511 | 0.0076 | 219.841 | 0.692 |  |
| Community & Hospital | sulfonamides_and_trimethoprim | linear | 27.003 | -8.5049 | 0.0062 | 271.078 | 0.684 |  |
| Community | sulfonamides_and_trimethoprim | exponential | 2.4971 | 0.3114 | 0.0041 | 94.2668 | 0.741 |  |
| Hospital | sulfonamides_and_trimethoprim | exponential | 2.6561 | -3.04 | 0.0396 | 78.1884 | 0.363 |  |
| Community & Hospital | sulfonamides_and_trimethoprim | exponential | 2.668 | -0.1016 | 4e-04 | 94.3744 | 0.918 |  |
| Community | sulphonamides_and_trimethoprim | linear | 37.4369 | -25.1028 | 0.1027 | 268.117 | 0.0901 |  |
| Hospital | sulphonamides_and_trimethoprim | linear | 24.8996 | -37.1962 | 0.014 | 219.693 | 0.591 |  |
| Community & Hospital | sulphonamides_and_trimethoprim | linear | 37.3596 | -22.7798 | 0.1033 | 268.098 | 0.0891 |  |
| Community | sulphonamides_and_trimethoprim | exponential | 3.169 | -0.963 | 0.0673 | 92.3653 | 0.174 |  |
| Hospital | sulphonamides_and_trimethoprim | exponential | 2.7091 | -3.3303 | 0.0513 | 77.9061 | 0.299 |  |
| Community & Hospital | sulphonamides_and_trimethoprim | exponential | 3.2552 | -1.0171 | 0.0917 | 91.5961 | 0.11 |  |
| Community | tetracyclines | linear | 35.5991 | -6.3597 | 0.0828 | 268.754 | 0.13 |  |
| Hospital | tetracyclines | linear | 27.7905 | -81.8174 | 0.041 | 219.055 | 0.354 |  |
| Community & Hospital | tetracyclines | linear | 35.7201 | -6.2548 | 0.0848 | 268.689 | 0.125 |  |
| Community | tetracyclines | exponential | 3.2038 | -0.2979 | 0.0809 | 91.9393 | 0.135 |  |
| Hospital | tetracyclines | exponential | 2.8281 | -5.2257 | 0.0765 | 77.2867 | 0.201 |  |
| Community & Hospital | tetracyclines | exponential | 3.2256 | -0.301 | 0.0875 | 91.7294 | 0.119 |  |
| Community | trimethoprim_and_derivatives | linear | 26.4597 | -20.8922 | 0.0743 | 269.022 | 0.153 |  |
| Hospital | trimethoprim_and_derivatives | linear | 24.5778 | -240.3826 | 0.0421 | 219.027 | 0.347 |  |
| Community & Hospital | trimethoprim_and_derivatives | linear | 25.9779 | -19.0488 | 0.069 | 260.803 | 0.177 |  |
| Community | trimethoprim_and_derivatives | exponential | 2.7834 | -1.0278 | 0.08 | 91.9664 | 0.137 |  |
| Hospital | trimethoprim_and_derivatives | exponential | 2.5717 | -9.8416 | 0.0323 | 78.3612 | 0.412 |  |
| Community & Hospital | trimethoprim_and_derivatives | exponential | 2.745 | -0.9213 | 0.0731 | 89.4462 | 0.164 |  |

The models in the above table can be compared by the R^2^ or the Akaike Information Criterion (AIC) value. However, as the latter is useful for predictions rather than model fit, we chose to use the R^2^ values to determine which model fit was “best”.

For linear model fits, there is a significant trend for 3G cephalosporins, beta-lactamase inhibitors and intermediate acting sulphonamides in the Community & Community and hospital, Community and hospital & hospital, and hospital respectively (figure S33). An exponential link is seen for more antibiotics (Figure S34): 2^nd^ generation cephalosporins, antibacterial for systemic, beta-lactams, beta-lactamase-resistant penicillins, beta-lactamase sensitive penicillins, glycopeptides, long-acting macrolides, macrolides, macrolides-lincosamides-streptogramins together and other beta lactams in a combination of different settings. The majority of significant interactions are driven by community use of antibiotics.

**Figure S33: Linear model fits with significant associations between use and MRSA incidence in different settings (top: community, middle: combined, bottom: hospital)**

**Figure S34: Linear model fits with significant associations between antibiotic use and log(base 10) MRSA incidence in different settings (top: community, middle: combined, bottom: hospital).**

**REFERENCES**

[1] Kime L, Randall CP, Banda FI, Coll F, Wright J, Richardson J, et al. Transient silencing of antibiotic resistance by mutation represents a significant potential source of unanticipated therapeutic failure. MBio 2019;10. https://doi.org/10.1128/mBio.01755-19.

[2] Li H. Aligning sequence reads, clone sequences and assembly contigs with BWA-MEM 2013.

[3] Stamatakis A. RAxML version 8: A tool for phylogenetic analysis and post-analysis of large phylogenies. Bioinformatics 2014;30:1312–3. https://doi.org/10.1093/bioinformatics/btu033.

[4] Seemann T. Shovill v1.0.9 2020. https://github.com/tseemann/shovill.

[5] Seemann T. Abricate 2020. https://github.com/tseemann/abricate.

[6] Seemann T. Prokka: rapid prokaryotic genome annotation. Bioinformatics 2014;30:2068–9. https://doi.org/10.1093/BIOINFORMATICS/BTU153.

[7] Page AJ, Cummins CA, Hunt M, Wong VK, Reuter S, Holden MTG, et al. Roary: rapid large-scale prokaryote pan genome analysis. Bioinformatics 2015;31:3691–3. https://doi.org/10.1093/BIOINFORMATICS/BTV421.

[8] Lees JA, Galardini M, Bentley SD, Weiser JN, Corander J. pyseer: a comprehensive tool for microbial pangenome-wide association studies. Bioinformatics 2018;34:4310–2. https://doi.org/10.1093/BIOINFORMATICS/BTY539.

[9] Jaillard M, Lima L, Tournoud M, Mahé P, van Belkum A, Lacroix V, et al. A fast and agnostic method for bacterial genome-wide association studies: Bridging the gap between k-mers and genetic events. PLOS Genet 2018;14:e1007758. https://doi.org/10.1371/JOURNAL.PGEN.1007758.

[10] The European Committee on Antimicrobial Susceptibility Testing (EUCAST). Antimicrobial susceptibility testing EUCAST disk diffusion method Version 7.0. 2019. https://www.eucast.org/ast_of_bacteria/previous_versions_of_documents.

[11] Rasigade JP, Barbier M, Dumitrescu O, Pichat C, Carret G, Ronnaux-Baron AS, et al. Strain-specific estimation of epidemic success provides insights into the transmission dynamics of tuberculosis. Sci Rep 2017;7. https://doi.org/10.1038/srep45326.

[12] Wirth T, Bergot M, Rasigade J-P, Pichon B, Barbier M, Martins-Simoes P, et al. Niche specialization and spread of Staphylococcus capitis involved in neonatal sepsis. Nat Microbiol 2020;5:735–45. https://doi.org/10.1038/s41564-020-0676-2.

[13] Wirth T, Wong V, Vandenesch F, Rasigade JP. Applied phyloepidemiology: Detecting drivers of pathogen transmission from genomic signatures using density measures. Evol Appl 2020;13:1513–25. https://doi.org/10.1111/eva.12991.

[14] Kuznetsova A, Brockhoff PB, Christensen RHB. lmerTest Package: Tests in Linear Mixed Effects Models. J Stat Softw 2017;82. https://doi.org/10.18637/jss.v082.i13.

[15] R Core Team. R: A language and environment for statistical computing 2017.

[16] Rasigade J-P. Time-scaled haplotypic density (THD) 2020. https://github.com/rasigadelab/thd.

[17] Cassini A, Högberg LD, Plachouras D, Quattrocchi A, Hoxha A, Simonsen GS, et al. Attributable deaths and disability-adjusted life-years caused by infections with antibiotic-resistant bacteria in the EU and the European Economic Area in 2015: a population-level modelling analysis. Lancet Infect Dis 2019;19:56–66. https://doi.org/10.1016/S1473-3099(18)30605-4.

[18] Rasigade JP, Leclère A, Alla F, Tessier A, Bes M, Lechiche C, et al. Staphylococcus aureus CC30 lineage and absence of sed, j,r-harboring plasmid predict embolism in infective endocarditis. Front Cell Infect Microbiol 2018;8. https://doi.org/10.3389/fcimb.2018.00187.

[19] Diep BA, Gill SR, Chang RF, Phan TH, Chen JH, Davidson MG, et al. Complete genome sequence of USA300, an epidemic clone of community-acquired meticillin-resistant Staphylococcus aureus. Lancet 2006;367:731–9. https://doi.org/10.1016/S0140-6736(06)68231-7.

[20] Monecke S, Slickers P, Ehricht R. Assignment of Staphylococcus aureus isolates to clonal complexes based on microarray analysis and pattern recognition. FEMS Immunol Med Microbiol 2008;53:237–51. https://doi.org/10.1111/j.1574-695X.2008.00426.x.

[21] Glaser P, Martins-Simões P, Villain A, Barbier M, Tristan A, Bouchier C, et al. Demography and Intercontinental Spread of the USA300 Community-Acquired Methicillin-Resistant Staphylococcus aureus Lineage. MBio 2016;7:e02183-15. https://doi.org/10.1128/mBio.02183-15.

[22] Ward JH. Hierarchical Grouping to Optimize an Objective Function. J Am Stat Assoc 1963;58:236–44. https://doi.org/10.1080/01621459.1963.10500845.

[23] Werkgroep Infectiepreventie (WIP). Meticilline-resistente Staphylococcus aureus (MRSA) 2012. https://www.rivm.nl/documenten/wip-richtlijn-mrsa-ziekenhuizen (accessed February 20, 2020).

[24] Lekkerkerk WSN, Sande-Bruinsma N van de, van der Sande MAB, Tjon-A-Tsien A, Groenheide A, Haenen A, et al. Emergence of MRSA of unknown origin in the Netherlands. Clin Microbiol Infect 2012;18:656–61. https://doi.org/10.1111/j.1469-0691.2011.03662.x.

[25] Lekkerkerk WSN, Haenen A, van der Sande MAB, Leenstra T, de Greeff S, Timen A, et al. Newly identified risk factors for MRSA carriage in The Netherlands. PLoS One 2017;12:e0188502. https://doi.org/10.1371/journal.pone.0188502.

[26] Leopold SR, Goering R V., Witten A, Harmsen D, Mellmann A. Bacterial whole-genome sequencing revisited: Portable, scalable, and standardized analysis for typing and detection of virulence and antibiotic resistance genes. J Clin Microbiol 2014;52:2365–70. https://doi.org/10.1128/JCM.00262-14.

[27] Knight GM, Budd EL, Whitney L, Thornley A, Al-Ghusein H, Planche T, et al. Shift in dominant hospital-associated methicillin-resistant Staphylococcus aureus (HA-MRSA) clones over time. J Antimicrob Chemother 2012;67:2514–22. https://doi.org/10.1093/jac/dks245.

[28] Toleman MS, Reuter S, Jamrozy D, Wilson HJ, Blane B, Harrison EM, et al. Prospective genomic surveillance of methicillin-resistant
 Staphylococcus aureus (MRSA) associated with bloodstream infection, England, 1 October 2012 to 30 September 2013. Euro Surveill
 2019;24:1800215. https://doi.org/10.2807/1560-7917.ES.2019.24.4.1800215.

[29] Hadfield J, Croucher NJ, Goater RJ, Abudahab K, Aanensen DM, Harris SR. Phandango: an interactive viewer for bacterial population
 genomics, Bioinformatics 2018;34:292–293. <https://doi.org/10.1093/bioinformatics/btx610>.

[30] Lindsay JA, Moore CE, Day NP, Peacock SJ, Witney AA, Stabler RA, Husain SE, Butcher PD, Hinds J. Microarrays reveal that each of the ten
 dominant lineages of Staphylococcus aureus has a unique combination of surface-associated and regulatory genes. J Bacteriol.
 2006;188:669-76. doi: 10.1128/JB.188.2.669-676.2006.

[31] McCarthy AJ, Lindsay JA. Genetic variation in Staphylococcus aureus surface and immune evasion genes is lineage associated: implications
 for vaccine design and host-pathogen interactions. BMC Microbiol. 2010;10:173. doi: 10.1186/1471-2180-10-173.

[32] Lindsay JA. Staphylococcus aureus genomics and the impact of horizontal gene transfer. Int J Med Microbiol. 2014;304:103-9. doi:
 10.1016/j.ijmm.2013.11.010.
