# Supplementary material for: Markers of epidemiological success of methicillin-resistant Staphylococcus aureus isolates in European populations
Source: Clin Microbiol Infect. 2023 Sep;29(9):1166–73. doi: 10.1016/j.cmi.2023.05.015 (PMC10775016; doi:10.1016/j.cmi.2023.05.015)
Supplement: Multimedia component 2 [file mmc2.pptx]

## Slide 1
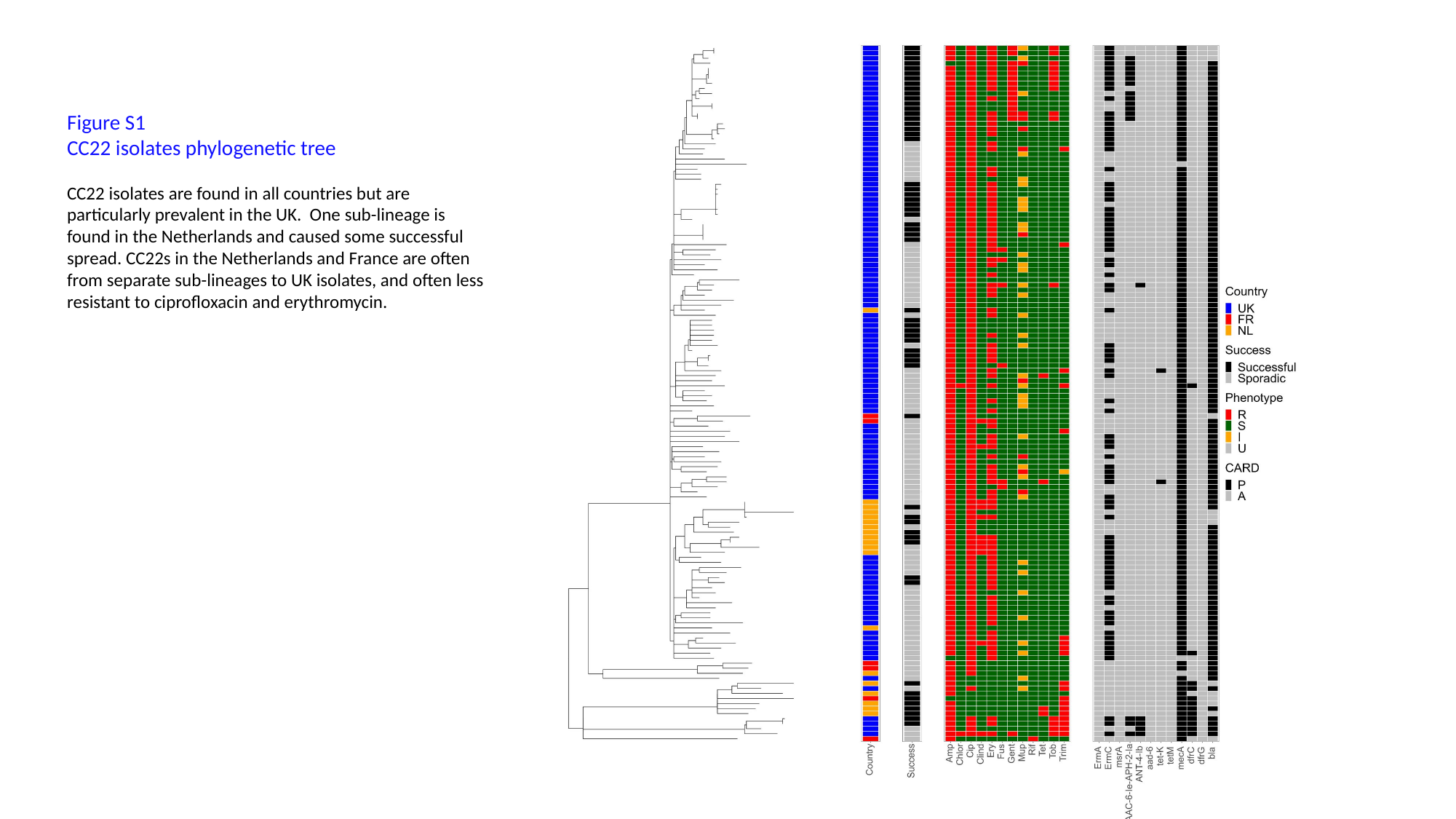

Figure S1
CC22 isolates phylogenetic tree
CC22 isolates are found in all countries but are particularly prevalent in the UK. One sub-lineage is found in the Netherlands and caused some successful spread. CC22s in the Netherlands and France are often from separate sub-lineages to UK isolates, and often less resistant to ciprofloxacin and erythromycin.

## Slide 2
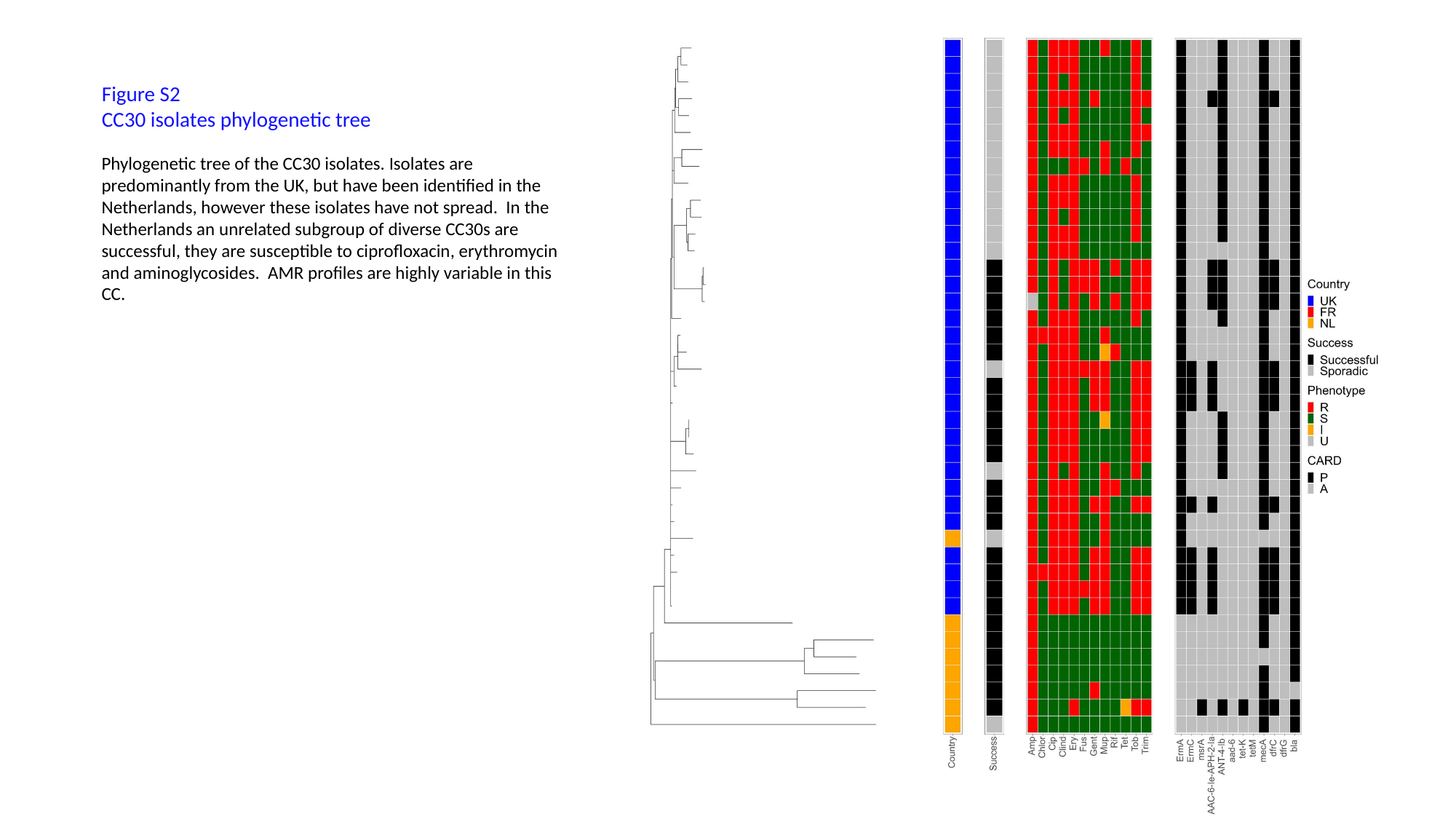

Figure S2
CC30 isolates phylogenetic tree
Phylogenetic tree of the CC30 isolates. Isolates are predominantly from the UK, but have been identified in the Netherlands, however these isolates have not spread. In the Netherlands an unrelated subgroup of diverse CC30s are successful, they are susceptible to ciprofloxacin, erythromycin and aminoglycosides. AMR profiles are highly variable in this CC.

## Slide 3
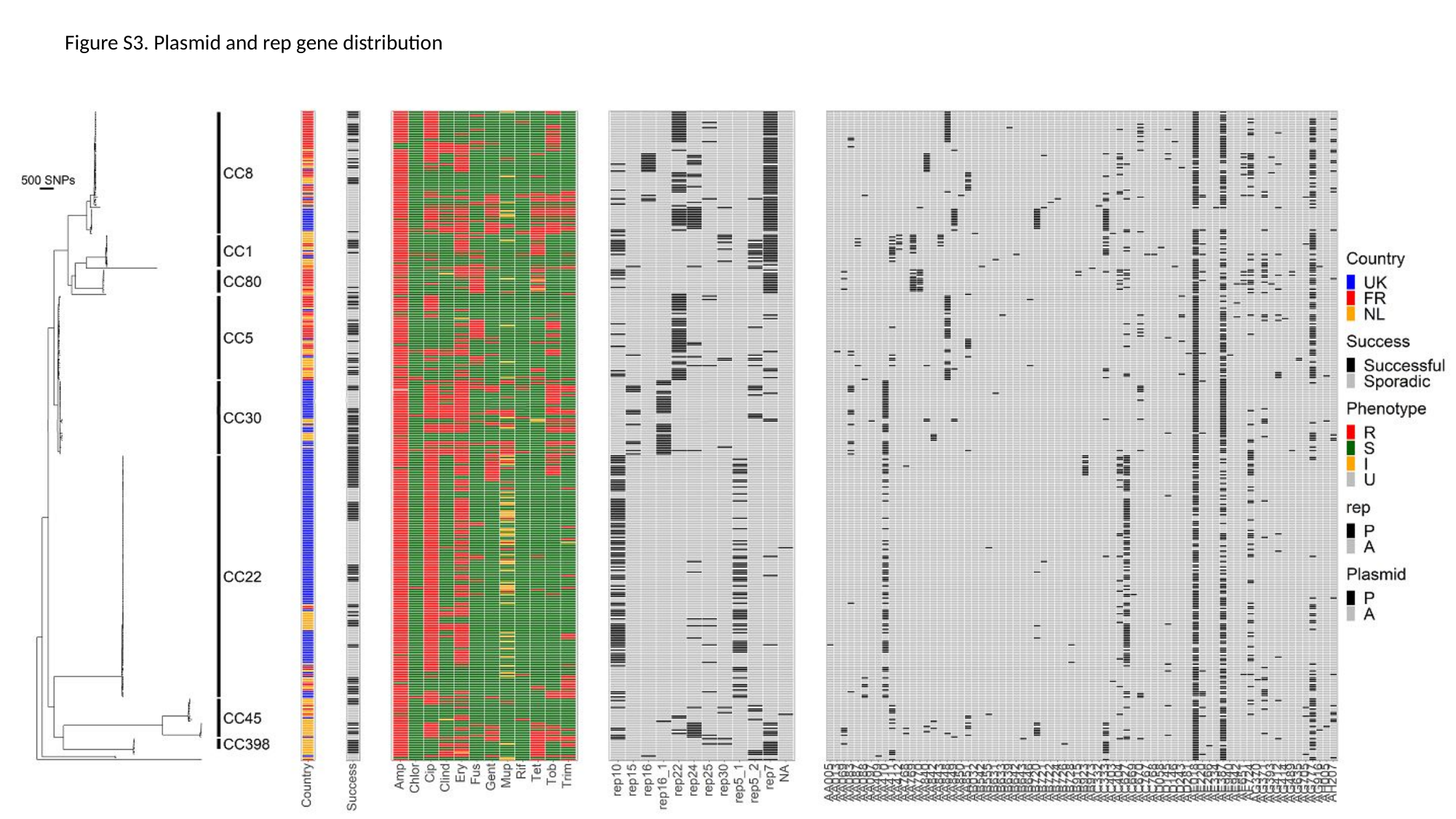

Figure S3. Plasmid and rep gene distribution

## Slide 4
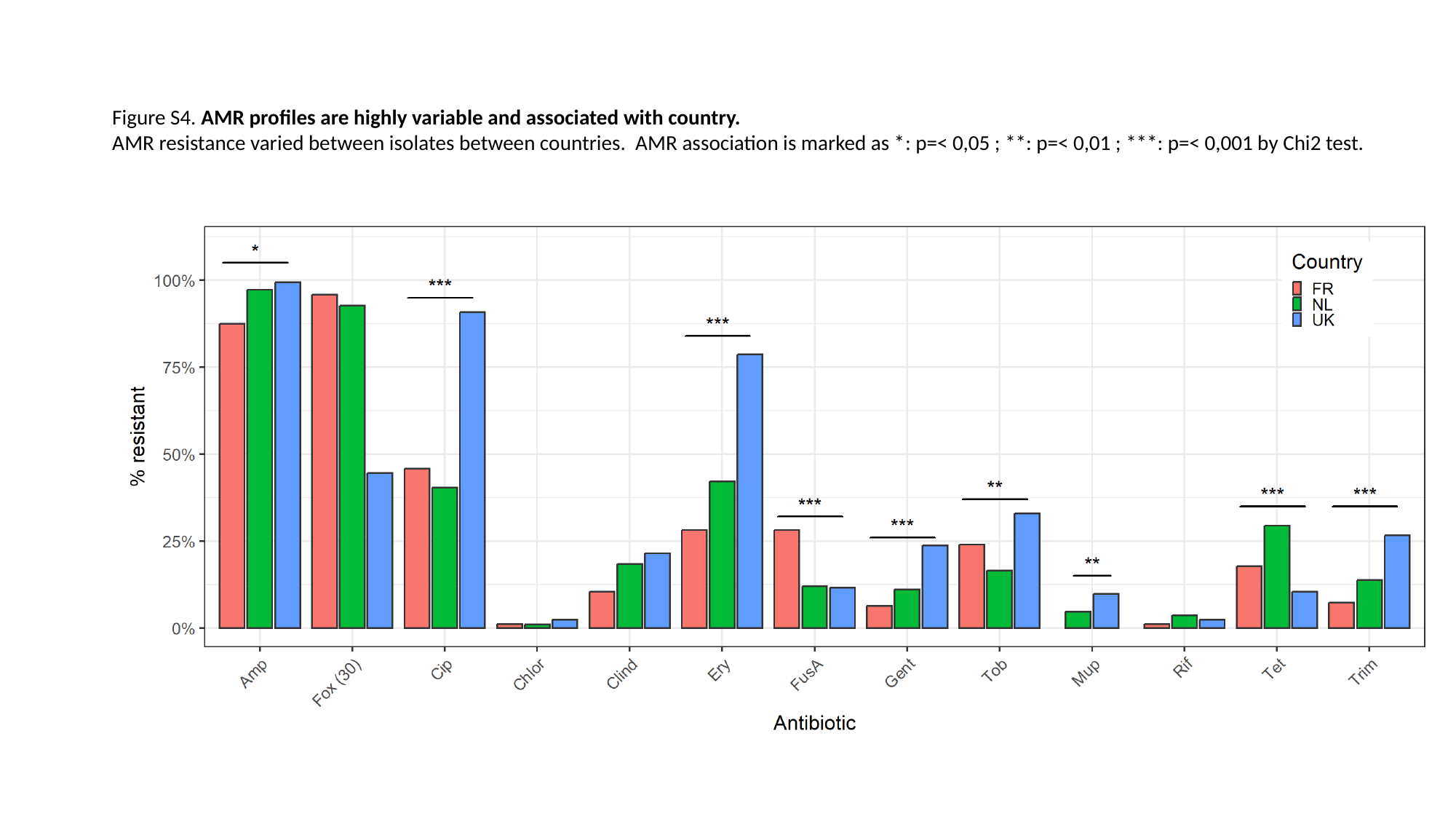

Figure S4. AMR profiles are highly variable and associated with country.
AMR resistance varied between isolates between countries. AMR association is marked as *: p=< 0,05 ; **: p=< 0,01 ; ***: p=< 0,001 by Chi2 test.

## Slide 5
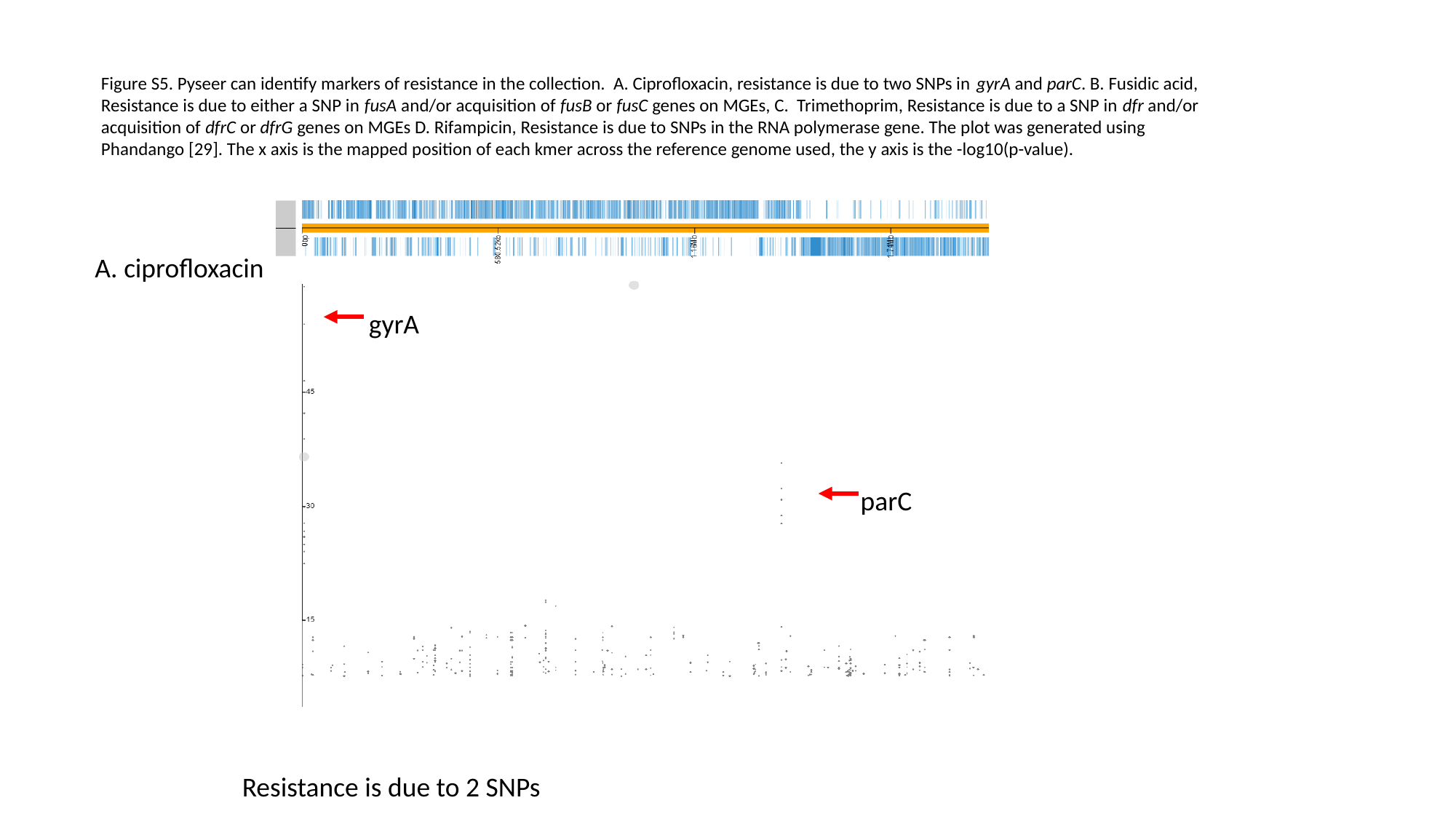

Figure S5. Pyseer can identify markers of resistance in the collection. A. Ciprofloxacin, resistance is due to two SNPs in gyrA and parC. B. Fusidic acid, Resistance is due to either a SNP in fusA and/or acquisition of fusB or fusC genes on MGEs, C. Trimethoprim, Resistance is due to a SNP in dfr and/or acquisition of dfrC or dfrG genes on MGEs D. Rifampicin, Resistance is due to SNPs in the RNA polymerase gene. The plot was generated using Phandango [29]. The x axis is the mapped position of each kmer across the reference genome used, the y axis is the -log10(p-value).
A. ciprofloxacin
gyrA
parC
Resistance is due to 2 SNPs

## Slide 6
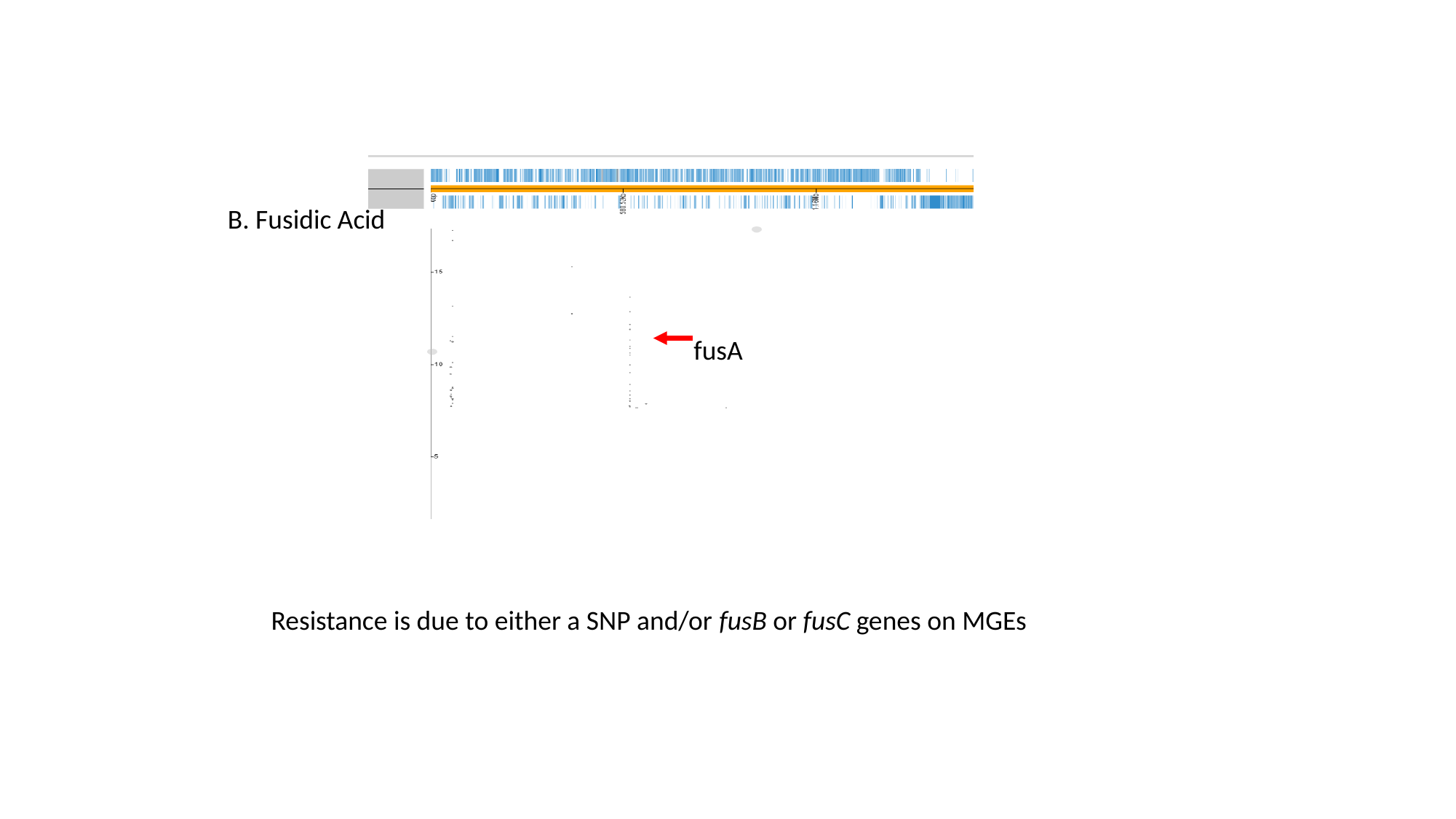

B. Fusidic Acid
fusA
Resistance is due to either a SNP and/or fusB or fusC genes on MGEs

## Slide 7
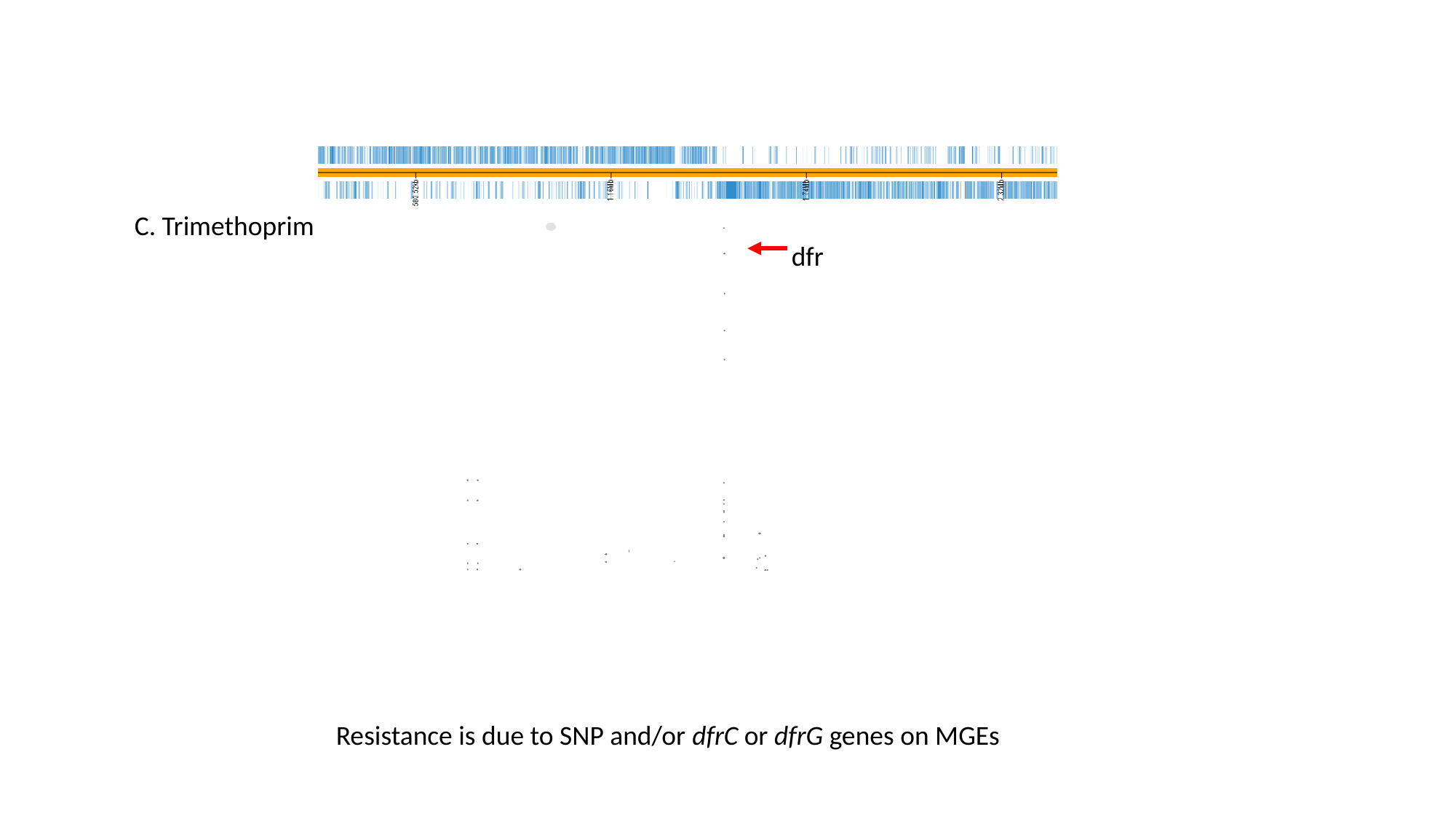

C. Trimethoprim
dfr
Resistance is due to SNP and/or dfrC or dfrG genes on MGEs

## Slide 8
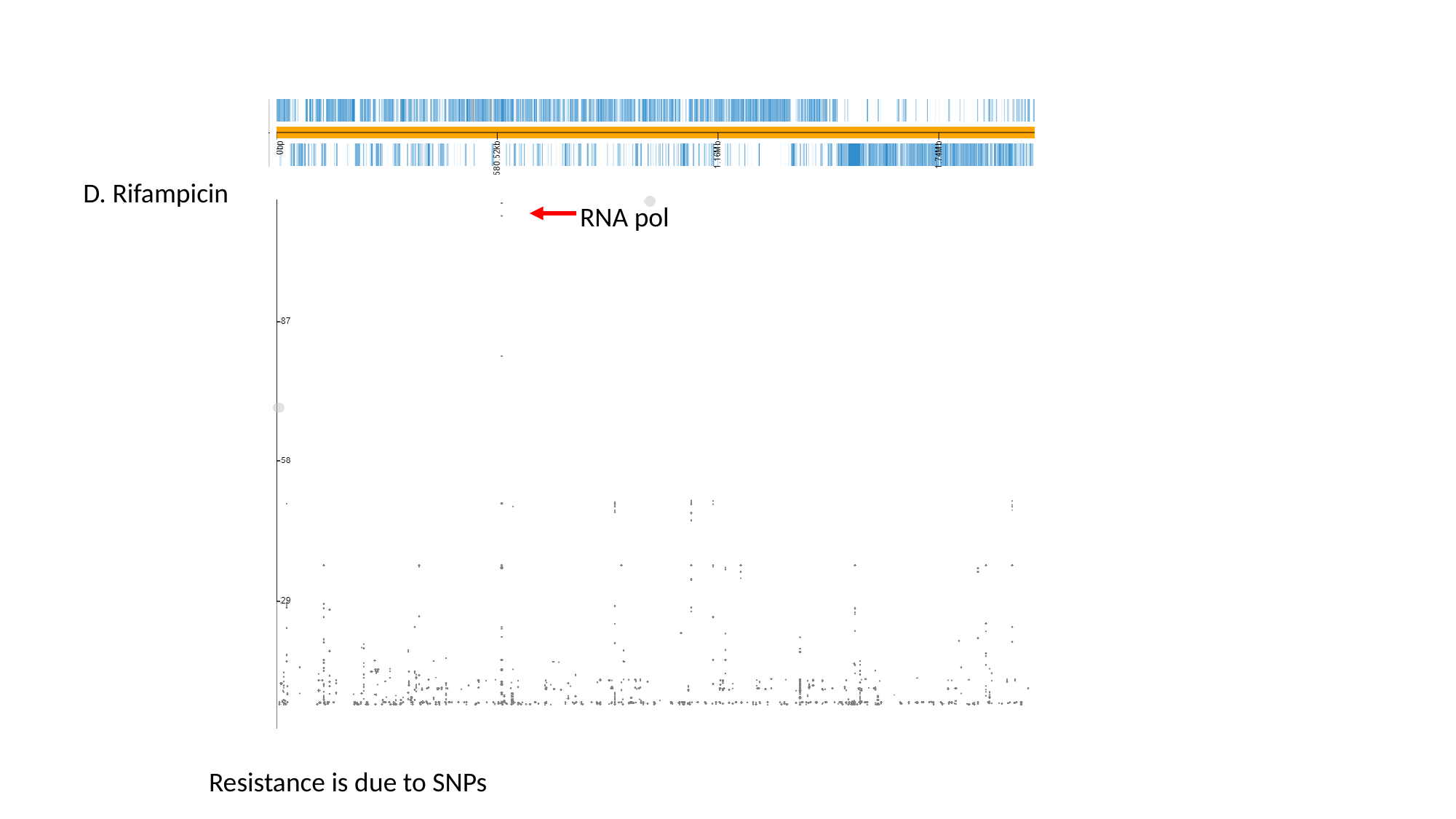

D. Rifampicin
RNA pol
Resistance is due to SNPs
